# High-Throughput Task to Study Memory Recall During Spatial Navigation in Rodents

**DOI:** 10.3389/fnbeh.2020.00064

**Published:** 2020-05-15

**Authors:** Lucia Morales, David P. Tomàs, Josep Dalmau, Jaime de la Rocha, Pablo E. Jercog

**Affiliations:** ^1^Institut d’Investigacions Biomèdiques August Pi i Sunyer, Barcelona, Spain; ^2^Neuroimmunology Program, IDIBAPS-Hospital Clinic, Universitat de Barcelona, Barcelona, Spain; ^3^Department of Neurology, University of Pennsylvania, Philadelphia, PA, United States; ^4^Catalan Institution for Research and Advanced Studies, Barcelona, Spain

**Keywords:** spatial navigation and memory, correlation between neuronal activity and behavior, single-session memory test, freely-moving calcium imaging recordings, data output for machine-learning algorithms analysis tools, high-throughput experimentation

## Abstract

Spatial navigation is one of the most frequently used behavioral paradigms to study memory formation in rodents. Commonly used tasks to study memory are labor-intensive, preventing the simultaneous testing of multiple animals with the tendency to yield a low number of trials, curtailing the statistical power. Moreover, they are not tailored to be combined with neurophysiology recordings because they are not based on overt stereotyped behavioral responses that can be precisely timed. Here we present a novel task to study long-term memory formation and recall during spatial navigation. The task consists of learning sessions during which mice need to find the rewarding port that changes from day to day. Hours after learning, there is a recall session during which mice search for the location of the memorized rewarding port. During the recall sessions, the animals repeatedly poke the remembered port over many trials (up to ∼20) without receiving a reward (i.e., no positive feedback) as a readout of memory. In this task, mice show memory of port locations learned on up to three previous days. This eight-port maze task requires minimal human intervention, allowing for simultaneous and unsupervised testing of several mice in parallel, yielding a high number of recall trials per session over many days, and compatible with recordings of neural activity.

## Introduction

### Relevance in the Study of Spatial Navigation

Successful navigation is crucial for survival and requires the memorization of locations and paths. It is believed that the hippocampus registers relevant events creating episodic memories that are stored in a mental structure called a “cognitive map” (Tolman, 1948). The discovery by O’Keefe and Dostrovsky (1971) of spatially tuned cells in the hippocampus (i.e., place-cells), gave support to the idea that the cognitive map internally represents in space (mostly) and time, events remembered during navigation (O’Keefe and Nadel, 1978). Based on this interpretation, the hippocampal spatially informative neuronal representation became the major focus of most investigations, leaving unattended the study of the hippocampal role in other types of memories (for review see Eichenbaum et al., 1999; Lisman et al., 2017) such as memories involved in time, stimulus-response associations, or rules over actions as a response to a given stimulus (McKenzie et al., 2014; Miller et al., 2017). Our task intends to investigate different components of mnemonic objects.

### Previous Long-Term Spatial Memory Tests

The tasks most widely used to study spatial navigation and spatial memories are the radial-arm maze (RAM) (Olton and Samuelson, 1976) and the Morris water maze (MWM) (Morris, 1981). The RAM tests long-term memories by measuring if the animal returns to the arm that was baited during a learning session held a few minutes, hours, or even days earlier (Chrobak et al., 2008). One disadvantage of the RAM test is that the number of trials per session is restricted to 6 or 8, limiting the ability to carry out a large number of repetitions needed to robustly assess the strength of the memory in a single session. In the MWM animals are put in a pool and have to remember the location of a platform that is removed after the learning trials, allowing for several independent trials for memory testing (4–8 trials per session). As a downside, MWM is limited in both the number of trials that rodents can perform per session and the difficulty of simultaneously recording neural activity. Alternative tasks (Barnes, 1979; Post et al., 2011), or modifications of the RAM and MWM (Bimonte et al., 2000; Rondi-Reig et al., 2006; Fouquet et al., 2013; Rossato et al., 2018, among others), have been used with success but also containing also some of the previously mentioned limitations (for review see Sharma et al., 2010; Vorhees and Williams, 2014). Another widely used task to test spatial memory is the spatial object recognition (SOR) task (Ballarini et al., 2009). In this task, the animal is presented with two familiar objects, one of which has been displaced from the position it occupied during a learning session a few hours earlier. The memory readout is based on one long trial that measures the time spent with the displaced object relative to the fixed one. As this reflects a natural behavior, the animals do not need to be trained. As a downside, scoring the memory is relatively subjective, hard to automate, and unfeasible to use to compare neural activity with behavior due to the lack of trial structure. In addition, some of the spatial navigation tasks that are currently used to link neuronal activity with behavior, have several caveats (Dupret et al., 2010; Pfeiffer and Foster, 2013) including labor-intensive human intervention, and a lack of overt behavioral memory responses that can be precisely timed, hindering the use of these tasks’ output data with machine learning methods to correlate neuronal activity with behavior, one of the main goals of our task.

Here we introduce a novel behavioral task with an automated pre-training phase of 3 to 4 weeks that is fully computer-controlled. This provides high-throughput data sets that yield robust statistical power. Once learned, the task is based on a trial structure wherein for each trial the animal starts from a random initial condition in the maze and learns to find the location of the reward. Our design forces the use of hippocampal-dependent spatial navigation with other sensory and internal representations stored in long-term memory. During the recall session, animals perform up to 20 trials during which they persist in trying to retrieve the remembered location. Moreover, the task can be repeated over dozens of sessions, easily yielding large amounts of data that allow investigation of memories stored long-term (e.g., several days). The task also allows the recording of neural activity without affecting the animal’s performance.

## Results

### The Task

**FIGURE 1:**
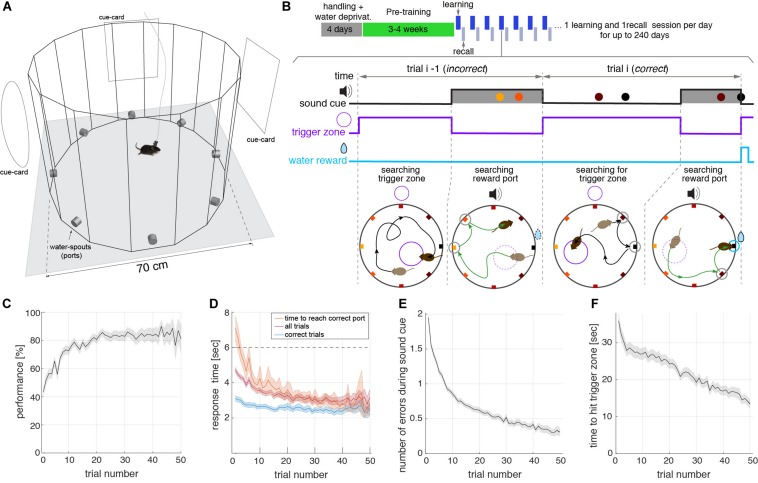
Trial structure of the spatial memory within a learning session. **(A)** Schematic of the open field built-in transparent acrylic with eight water spouts (ports) controlled by Python-based software coupled to Arduino boards. The arena is placed inside a 1 cubic meter sound isolation chamber with cards placed on the walls serving as distal visual cues for the animals. **(B)** Top: Schematic of the behavioral protocol, showing handling, pre-training, and actual-experiment phases. Learning and recall sessions are performed on a daily basis until the animal’s motivation persists over days. Bottom: Schematics of two consecutive trials during a learning session of the task. Top traces show the temporal structure of the sound, trigger zone activation, and the reward, along with the pokes in the different ports (colored dots). Bottom diagrams show the trajectory of the animals and the location of the ports in the maze. Ports are colored according to the distance to the correct port (black port at **E**). In the first part of each trial, animals walk around the box seeking to start the reward time window during which water is available (black trajectory in bottom panels). The reward time window lasting for a maximum of 6 s, starts when the animal steps into an invisible trigger zone, randomly placed in a different location on every trial (small violet circle in bottom diagrams). During this time window, a sound is played cueing the mouse about the availability of reward (green trajectory). If the animal pokes the correct port, the sound cue stops. If the animal does not reach the correct port during this window, perhaps because it poked in a non-rewarding port, the trial is considered incorrect (trial *i*−1 in **B**). In contrast, if the animal reaches the correct port, independently of whether it poked in incorrect ports before (typically close to the correct port), it receives the reward (10 μL of water), the sound stops, and the trial is considered correct (trial *i* in **B**). The correct port is fixed for each day (learning and recall sessions) but changes randomly from day to day. **(C)** Performance (i.e., correct over total trials) versus the trial number in learning sessions. All measures in **(C–F)** represent averages across all animals and sessions (*n* = 23 mice). Shaded areas represent a 95% confidence interval of the SEM. The performance in the first trial was higher than 1/8 because animals poked in multiple ports during the tone **(E)**, setting the probability to hit the correct port on the first trial above 40%. **(D)** Response time during learning sessions. Response time was defined as the interval from reward time window onset to nose poke in the correct port in three different conditions: for correct trials only (poke correct port before 6 s, blue), for correct and incorrect trials (magenta) (Note: incorrect trials response time was set to 6 s), and total time to reach the correct port (orange) even after the 6 s sound cue window. **(E)** The number of errors during the sound cue is the number of ports poked before poking the correct port in learning sessions. Errors decrease with the trial number. **(F)** Time to find trigger-zone in learning sessions. After the reward time window is finished, we computed the time from this moment until the animal hits the randomly placed trigger zone. We show here that animals improve navigation toward the correct port within each session.

The main goal for the design of the memory task presented here was to obtain a large number of trials during a spatial memory recall session with minimal human intervention. The task consisted of a spatial navigation search within a circular arena, to learn and then recall, the location of a rewarding water-spout (port) (Figure 1A). The behavioral box had transparent walls forming a hexadecagon, to allow for the visualization of distal cues. Located on the walls were eight equidistant ports that measured nose pokes and could deliver water. The control of the ports, as well as the real-time video tracking of the animal, was monitored by computer software developed by the authors. The task did not require any human intervention other than moving the animals in and out of the box and initiating the behavior acquisition program. The behavioral box was installed in a sound isolation chamber, which shielded the animal from sound arriving from the external room and from neighboring chambers. The one cubic meter chamber also had distal visual cues, in the form of cards, located on its inner walls (Figure 1A). Moreover, the use of isolation chambers allowed the placement of several behavioral setups in the same room (see Supplementary Figure 1), making it scalable to a large number of experiments run in parallel. During the pre-training phase that lasted ∼3–4 weeks (Figure 1B, see section “Methods”) the animal learned the task, and then was followed by the actual experiment phase composed of two sessions: learning and recall. During the learning session, lasting 15 min, animals learned which was the rewarding port on that day. Rewarded ports were randomly selected for each animal and each day. The learning session started after each animal spent 100 s of acclimatization exploring the arena (see section “Methods”). To initiate the experiment animals sought to activate the reward availability by entering an invisible trigger zone: an invisible circle occupying a fraction of the arena’s area (1/16) that was randomly placed for each trial (Figure 1B). When the animal walked into the trigger zone, the reward window started, lasting up to 6 s while a sound cued the animal about the availability of water at the rewarding port. The cue was a pure tone [7040, 9060, 10560] Hz (different for each behavioral box) played at 40 dB above background noise. A trial was considered correct if the animal poked into the correct port during the reward time window, even if it poked in other ports prior to poking the correct port (see, e.g., *trial*-*i* in Figure 1B). The reward time window was interrupted when the water reward was harvested (see the correct poke closing the reward window in *trial i* in Figure 1B). Error pokes were not actively punished. Trials in which the animal did not poke the correct port during the reward window, were computed as incorrect trials (see *trial i-1* in Figure 1B, see also Supplementary Video).

Several aspects of the behavioral analysis demonstrated that animals quickly learned the location of the rewarded port during each individual learning session. Average performance, defined as the percentage of correct trials, rapidly increased with the number of trials until it reached a plateau of around 80–85% (Figure 1C). Animals reached 80% performance in less than 20 trials in a given learning session. The animals became faster in arriving at the correct port during the session (Figure 1D) obtaining the reward in approximately 2.5 s from the onset of the reward time window. As the session progressed, they also made fewer errors during the sound cue, indicating that their spatial accuracy within a session increased with the trial number (Figure 1E). The average time to find the trigger zone also decreased significantly along with the session (Figure 1F). Even though error pokes on-tone and off-tone were not punished, the time constraint of the trial structure and willingness to maximize water intake resulted in a decrease of error pokes during the tone and inter-trial period in all animals (Supplementary Figure 2). Altogether this shows that animals were able to learn the location of the rewarded port on a given day and then use that information to maximize the water intake.

### Demonstrating Hippocampal Dependency on the Task

An important aspect of the task is that animals were forced to navigate in space by starting each individual trial from a different location due to the random placement of the trigger zone (Figure 1B). Randomly distributed trigger zones forced the animals to initiate the run toward the rewarded port from a different position and with a different orientation and body posture relative to the correct port on each trial (Supplementary Figure 3a) inducing allocentric spatial navigation. In addition, the task provided a dense spatial coverage of the arena (Supplementary Figure 3b), a requisite for a good characterization of neural activity encoding spatial information (e.g., place-, head- direction-, and grid-cells). To determine if the animals were relying on odor cues, several times during the session we removed the animal from the arena, cleaned the surfaces of the maze and individual ports with water and mildly scented soap. The animals did not show changes in their navigation accuracy, in comparison with control experiments in which the animals remained in the arena without cleaning (Supplementary Figure 4). We have also found that animals did not display biases with respect to any particular port or spatial location (data not shown).

In spatial navigation tests, animals can use stereotypical trajectories to solve the task which does not rely on spatial memory. When stereotypical trajectories are used there should be a strong dependence of the performance on the initial position of the animal relative to the correct port. We found that performance did not depend significantly on the location of the initiation point relative to the rewarding port (Supplementary Figures 5a–d) or on the heading-direction angle at the initiation point relative to the rewarding port (Supplementary Figure 5e). Therefore animals did not use a stereotypical strategy to solve the task, but rather their strategy was flexible enough to find the rewarded port in a diversity of conditions imposed by the randomization of trigger zone location.

As a further demonstration that the hippocampus is necessary to solve the task, muscimol (at a dose that did not affect locomotion) or saline were injected intracranially aiming to the dorsal hippocampal area, on alternating days in the same animal. The performance on the days when muscimol was injected was significantly lower than when saline was given (Supplementary Figure 6). This supports the requirement of the dorsal hippocampus to solve the task.

### Detailed Analysis of Animal Behavior During the Task

**FIGURE 2:**
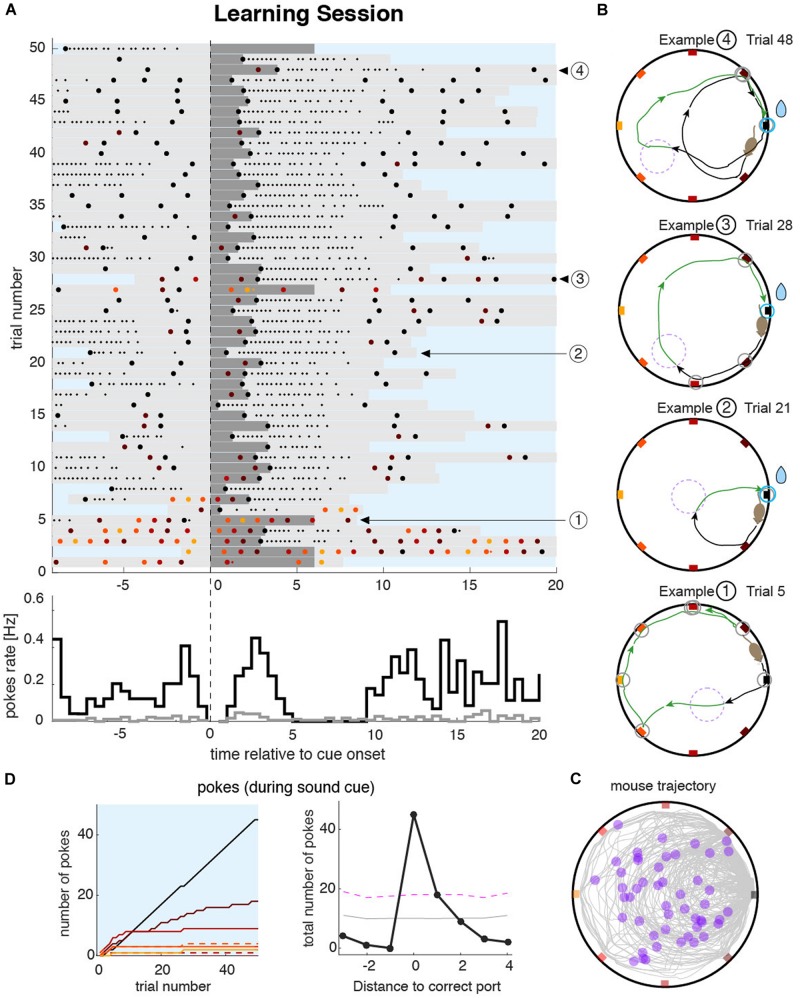
Mouse behavior during the learning sessions. **(A)** Top: raster plot of pokes during an example learning session shows the timing of pokes at different ports (colored dots matching the port color code) ordered in time by the sound cue onset on each trial (vertical dashed line). Dark gray bars represent the sound cue duration (as in Figure 1D magenta line). Light gray bars indicate the duration of the trigger zone seeking phase of the trial (as in Figure 1F). Small dots indicate persistent licking in a given port. In this example session, the animal sought the correct port during the first five trials after which the animal started to accurately find it in almost all consecutive trials. Bottom: pokes cue-triggered time histograms for the correct port (black line) and the average overall incorrect ports (gray line). The time bin for the poke rate is 0.5 s. **(B)** Trajectories during four example trials. The color code of pokes and trajectory parts are the same as in Figure 1B. Trials at the beginning of the session were more explorative (Example 1) until the animal unequivocally identified the correct port location (e.g., in trials 6–9 in this example). From that point on, trajectories were either directed toward the correct port (Example 2), or with only one error port during the sound cue (Examples 3 and 4). **(C)** All trajectories from all trials accumulated over the entire learning session. Violet dots represent the position of the animal at sound cue onset. **(D)** Left: cumulative poke count vs. trial number for each individual port during sound cue. The correct port (black line) shows a higher poke count than any other port during the tone. Pokes on different ports are plotted with color code as in **(A–C)**. Dashed lines indicate the counterpart port at equal distance from the correct port. Right: poke histogram during sound cue, for each port index ordered by the distance to the correct port. Magenta dashed line represents the significance level (*P* < 0.01) over which poking probability was significantly larger than that expected from a uniform distribution. Gray dashed line is the mean value of the uniformly shuffled data.

We capitalized on the features of the task that force the animals to make overt and discrete responses in order to obtain the reward. We used the spatial distribution and timing of pokes to characterize the behavior during the learning sessions. Poking patterns in each session could be visualized by drawing poke raster plots (Figure 2A). During the training session, the goal of the animal is to identify the rewarding port as fast as possible. To achieve this, the animal started the learning session by poking at the maximum number of ports during the sound cue (Figure 2A bottom of the raster plot; see also Example 1 in Figure 2B). Once the reward was obtained after a few trials (e.g., 2–3), the animals generated trajectories with almost no errors (Example 2 in Figure 2B). When they made errors, these were typically on the closest port relative to the rewarding port (Examples 3 and 4 in Figure 2B). The complete trajectory and locations of the trigger zones are plotted in Figure 2C, illustrating the diversity of the trajectory shapes from a single session. To summarize the poking statistics during the entire session, we built poke histograms (Figure 2D, right). The narrower the poke histogram around the correct port, the more spatially precise was the animal at solving the task. This allowed for detailed quantification of the accuracy of the animal’s navigation and memory, a feature we also exploited in the quantification of the memory accuracy during recall sessions.

**FIGURE 3:**
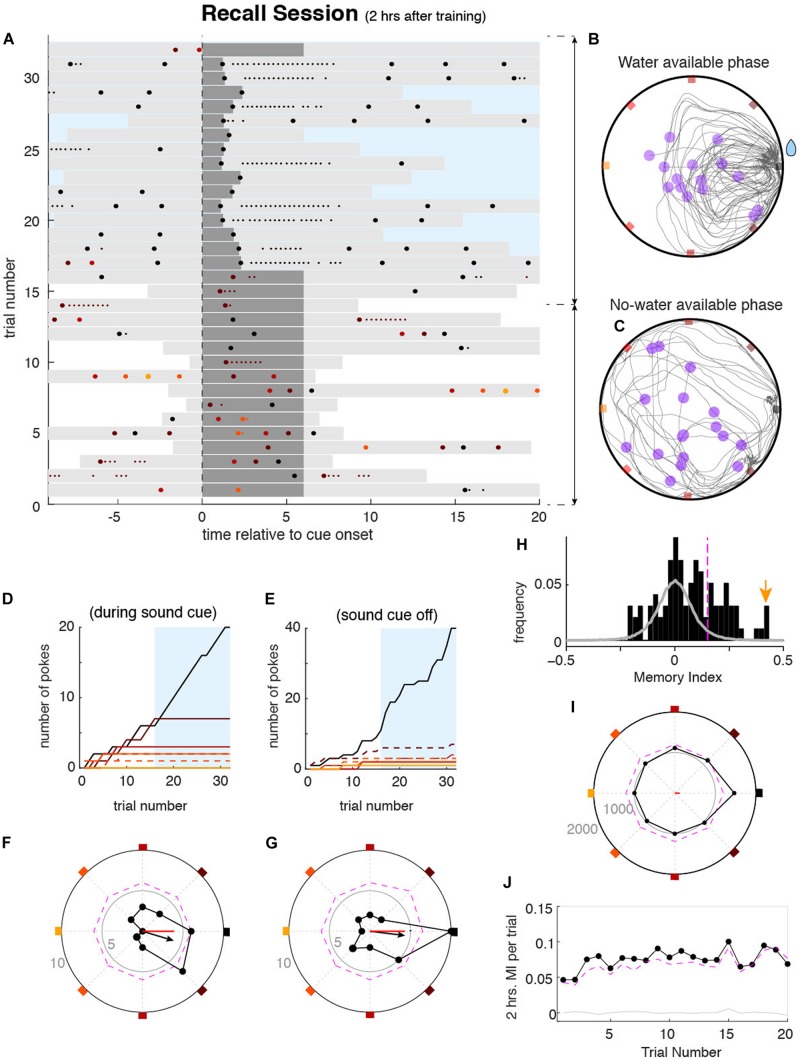
Mouse behavior during the memory recall sessions. **(A)** Pokes raster plot during an example memory recall session performed by mouse 4032, displayed as explained in Figure 2A. During recall sessions, correct pokes during the first *n* minutes of the session (*n* = 0, 1, 3, or 5) were not rewarded, to avoid re-learning of the reward location. Sound cues were never interrupted during this period. After the *n* minutes, correct pokes were rewarded as during the learning session (blue rectangle). In this example session, during the first *n* = 5 min, corresponding to trials 1–16, water was not available, whereas in trials 17–31 water was available. **(B,C)** All trajectories from all trials accumulated over the recall session for the period without water **(F)** and water **(G)**. Representation is equivalent to that shown in Figure 2C. Cumulative poke count versus trial number for each port during sound cue **(D)** and sound cue off **(E)**. Correct port (black) and one neighbor port (dashed brown) show higher poke count in both cue on and cue off conditions during the period without water. Once the water was available and harvested (blue area), the rewarded port was mostly poked. Poke histograms in polar coordinates for the example session (black dots), showing the number of pokes in each port as the radial distance of each point to the center, for the cue on **(F)** and cue off **(G)** conditions. Colored squares mark the angle of each port (as in Figure 1B). Small gray numbers show the count number of the radius of the inner and outer circles. The dashed magenta line shows the pointwise significance bound obtained from shuffled data (*P* < 0.01, one-tailed; see section “Methods”). Black arrows show the vector summation of the counts over all the ports. Red lines show the MI for the session relative to the 2 h correct port. **(H)** Histogram of MIs obtained from individual recall sessions of mouse 4032 (black bars). Gray histogram shows the MI values from the surrogate data set drawn from a uniform poking distribution. The magenta vertical line shows the significance bound of individual session MI (*P* < 0.01). The yellow arrow marks the session shown in **(A–G)**. **(I)** Accumulated poke histogram for all sessions (*n* = 127) of mouse 4032 considering both on and off cue conditions. Magenta dashed line indicates significance bound (*P* < 0.01, one-tailed). The red line shows the MI over all sessions. **(J)** 2 h average MI versus trial index during the recall session. Despite the absence of water during these trials, MI was significantly larger than zero over the first 16 trials, illustrating the persistence of the animal to recall the rewarding port.

Two hours after the learning session, animals performed a recall session of 10 min to measure if they remembered the rewarding port. Two hours were chosen because prior studies have shown that this is the minimum time needed for NMDA-dependent memory consolidation to occur (Kentros et al., 1998; Steele and Morris, 1999). We also tested memory at 3 and 4 h delays to determine if the deficit in memory consolidation induced by the blockade of NMDA-r was maintained, and found no significant difference with the 2 h delay results (data not shown). During the first part of the recall session, the reward availability was randomly set across days and was immediately available or delayed by 1, 3, or 5 min. Additionally, during the delay, the sound cue did not stop when they poked the correct port (Figure 3A). The absence of feedback was to prevent the animals from re-learning the position of the correct port. The randomness of the delay in the reward was to maintain the motivation of animals. Except for these modifications, the learning and recall task components were identical (for more details see section “Methods”). In these conditions of both spatial and temporal uncertainty, mice sought the reward during several trials (e.g., ∼10–20) (Figures 3B,C, see also below and Figure 3J). These trials were used as equivalent repeated memory probes. We then measured the strength of the spatial memory by quantifying the probability of pokes in the different ports (e.g., trials 1–16 in the example shown in Figure 3A). Despite the individual variability in their poking behavior, animals exhibited a significant tendency to preferentially poke the correct port learned during the learning session (Figures 3D,E). Once the reward became available, animals focused almost exclusively on poking the rewarded port without much behavioral variability (Figures 3A,B,D,E, light-blue areas) similar to the later phase of the learning sessions. This last part of the session was not included in the assessment of memory strength but contributed to the formation of long term memory, tested on consecutive days.

To quantify the recall accuracy for a particular stored memory, we created the *memory index* (MI), independent of the number of trials, total number of pokes, and length of time of the unrewarded period during the memory test. The MI could be computed from single sessions or averaged across sessions. To compute the MI, we first built a spatial poke histogram represented in Figures 3F,G with polar coordinates. Due to the circularity of the task, this choice of coordinates easily illustrated the tendency of the animal to poke the ports in each orientation of the box perimeter (as an example Figures 3F,G shows a tendency to poke the correct port and the closest neighbor on the S-E direction). We then defined the *pokes vector* by performing a vector sum with the contributions of pokes on each of the ports, each pointing in the direction of a different port, and normalized by the total number of pokes (see section “Methods”). The resulting vector summarized the probability to poke in a certain direction (see black arrow in Figures 3F,G). By projecting the vector onto the axis of any port we obtained the MI quantifying the net poking preference toward or away from that port (the reference port) (red line in panels Figures 3F,G). The MI ranged between + 1 (all pokes were in the reference port) and −1 (all pokes were in the port opposite the reference port). The MI was based on the poking probability in each of the ports and not exclusively on the reference port, which conferred to the MI a robust statistical description of animals’ behavior. Moreover, the MI was independent of the total number of pokes during the session, allowing the comparison across sessions and animals.

We first used the MI to quantify the accuracy of the memory of the port learned 2 h before each recall session, which became the reference port. In certain sessions, animals generated a 2 h memory response with high recall accuracy (Figures 3F,G). Across sessions, however, animals exhibited some variability in the MI (black histogram in Figure 3H). To test for significance of the MI in each session, we generated a distribution of MIs obtained from a surrogate data set, that lacked any spatial preference: the surrogate data had the same number of pokes per session as the original data but was generated following a uniform poking distribution across ports (gray-line histogram in Figure 3H). Using the 99% percentile of the shuffle distribution we computed the significance level at *P* < 0.01 (one-tailed; vertical dashed magenta line in Figure 3H; see section “Methods” for details). For example Figure 3H shows that 41 out of 127 recall sessions of one animal yielded a significant MI. Capitalizing on the design of the task, memory accuracy could also be quantified by pooling sessions performed on different days with different reference port for the MI. For this, we pooled the poke histograms from multiple individual sessions, each having the correct port oriented to NE, into one single poke histogram (Figure 3I shows animal 4032). Statistical significance was then assessed by shuffling the values of each individual session histogram and then summing across sessions to obtain a pooled shuffled histogram (dashed magenta line shows *P* < 0.01 level). Pooled poke histograms for individual mice are shown in Supplementary Figure 7. From the pooled histograms, a MI was obtained for each animal, as done from individual session histograms, where 17 out of 23 mice showed a significant MI for the data collected (*P* < 0.01, one-tailed permutation test). Our metric of recall accuracy, the MI, allowed us to demonstrate that the large majority of our animals were able to express significant recall (*P* < 0.01) of the port learned 2 h before when averaging across sessions (Supplementary Figure 7). We then asked whether the 2 h memory recall accuracy quantified by MI showed across-trial dynamics during the no water period of the recall session. If receiving no water upon poking the remembered port acts as negative feedback, inducing the mice to explore other ports, this would be reflected as a decrease of the MI. To measure the dynamics of memory recall during the no water period, we pooled all sessions for each animal and averaged across animals to compute the average MI at each trial during the no water period. We found that the tendency to poke on the remembered port was maintained even in the absence of the reward when pooling all data together; the MI was relatively constant and remained significant over the first 16 trials (*P* < 0.01, Figure 3J). This finding justifies our choice to compute the MI, based on all the trials during each session as they can be viewed as equivalent repeated memory probes.

### Assessing Memory Recall From Previous Days

**FIGURE 4:**
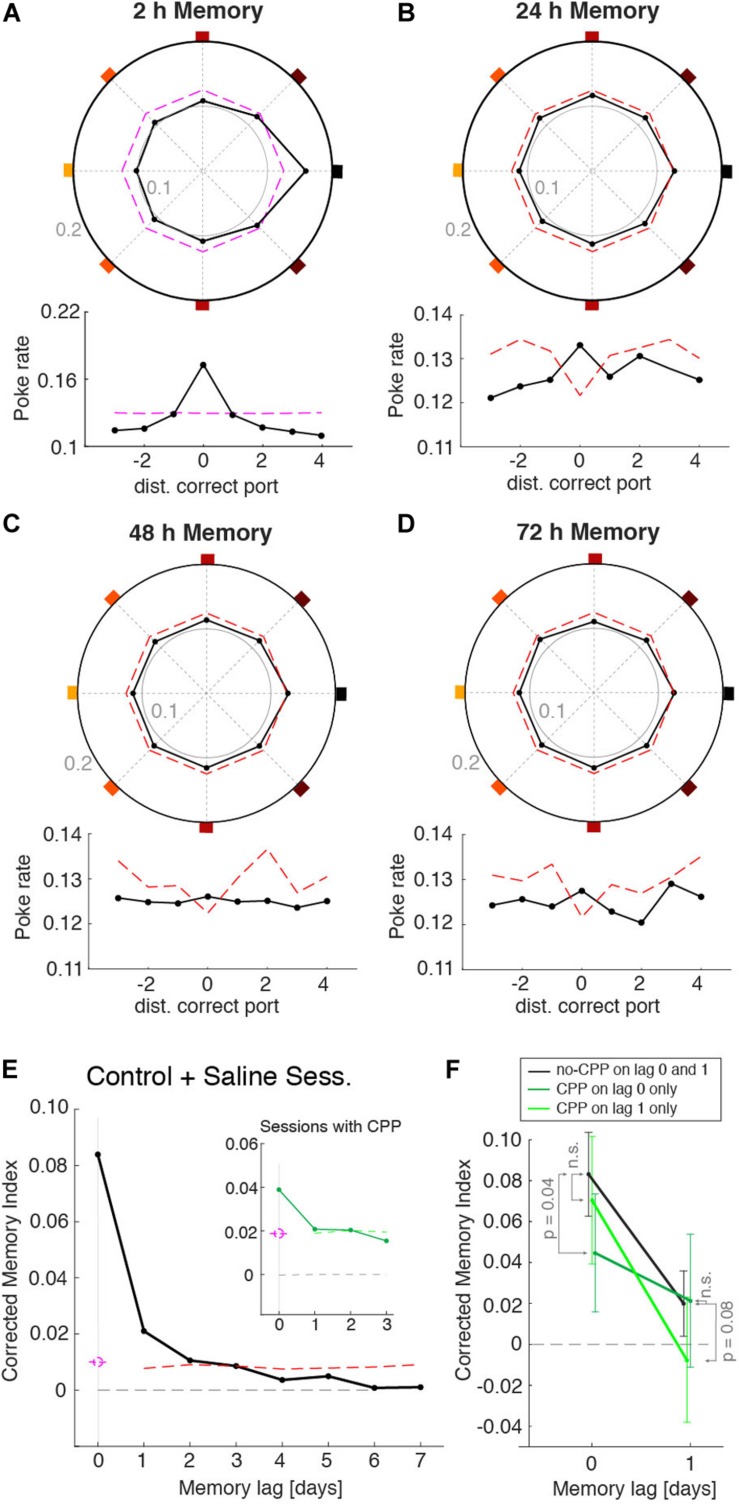
Memory recall from previous sessions. Normalized poke histograms aligned at the port learned 2 h **(A)**, 24 h **(B)**, 48 h **(C)**, and 72 h **(D)** before (black line + dots) show a significant preference to poke in the ports memorized in the three previous days (*P* < 0.01, permutation test). Histograms were the average across animals (*n* = 23 mice), each with a different number of sessions (mean no. sessions 55; range: 11–127). Lower insets show the same poke histograms unfolded for finer visualization. The red dashed line is the top part of the confidence interval of the surrogate data generated with the same 2 h memory strength contained in the real data. **(E)** Corrected memory index MI (MI – mean surrogate shuffles) versus the session lag (black dots, *n* = 23 mice) shows that there was significant memory recall of the correct ports from the four previous sessions (i.e., up to 72 h). Significance was assessed generating a surrogate data set with *only* 2 h memory that followed the same sequence of rewarded ports across days (mean across surrogates is shown in gray). Results from control (no injection) and saline injections sessions are combined here as they show no difference. **(F)** Averaged corrected MI for 2 and 24 h memories. Consecutive sessions were grouped in three conditions: (i) sessions with no CPP (black line), (ii) sessions with CPP injected only on the testing day (dark green line), and (iii) sessions with CPP injected only on the previous day (light green line). CPP decreased the MI only on the day that it was injected: MI decreased in lag 0 when CPP was injected on the test day (*P* < 0.05, one tail *t*-test) and in lag 1 when it was injected on the previous day (*P* = 0.08, one tail *t*-test). Lag and CPP conditions showed a significant interaction (*P* < 0.022, two-way ANOVA). Error bars are the 95% confidence interval.

Despite the ability of mice to remember the port that provided the reward during the learning session, their behavior during individual recall sessions was quite variable, having different dynamics during the non-rewarded periods. We thus asked whether this variability could be caused, at least in part, by the recall of the memories acquired on previous days. For instance, in the recall session shown in Figures 3F,G, in addition to the increased probability to poke the 2 h memory port (east port), this animal also showed a strong tendency to poke the S-E port (Figures 3F,G). It turned out that this port was the correct port on the previous day (24 h memory). Was this deviation toward ports learned on previous days systematic across animals and sessions? To answer this, we built a poke histogram for each animal by pooling all sessions but aligning the pokes of each session to the correct port learned *n* days ago (*n* = 0, 1, 2, 7). We then normalized these histograms and took the average across animals to maximize the statistical power (Figures 4A,D). As expected from the individual animals’ 2 h memory recall (Supplementary Figure 7), the MI for lag 0 days (i.e., 2 h) was significantly different from zero (*P* < 0.01). To assess the statistical significance of the MI of locations learned in previous sessions, we built 500 surrogate data sets, each composed of the same number of sessions and pokes as the original data set, yielding the same 2 h MI as the original data but in which, by construction, we eliminated any trace of previous memories in the surrogate data (i.e., 24 and 48 h; see section “Methods” for details). For each of these surrogate data sets, we calculated the MI of memories learned on previous days using the exact same across-day sequence of rewarded port indices used in the experiment. By construction, the MIs for previous days should be zero on average. However, the surrogates had a decreased probability to visit the rewarded ports from previous days, which yielded a negative average MI (Figures 4B–D). This was because the sequence of rewarded ports was generated from permutations of the eight indices to minimize the probability that the same port was rewarded in consecutive days. This weak negative correlation in the ports sequence (Supplementary Figure 8a) made that any increase in the probability to visit today’s rewarded port (i.e., 2 h MI > 0) automatically caused a decrease, compared to a uniform distribution, of the probability to visit previous session’s port (see dashed lines in Figures 4B–D and Supplementary Figure 8b). The average MI of the surrogate data for the memories from immediately preceding days was thus negative and was used as a baseline to which the MI of the original data could be compared. The corrected MI was thus defined as the difference between the original data and the mean of the surrogates (Figure 4E). From the distribution of corrected MIs obtained from the surrogate data we obtained a confidence level (99%, one-sided) to assess the significance of the MI (Figure 4E red dashed line). We found that the animal-averaged corrected MI was significant for 24 h (*P* < 0.01), 48 h (*P* < 0.01), and 72 h (*P* ∼ 0.01) (black dot over red dashed line in Figure 4E). For longer session lags, the MI was not statistically different than the surrogate data (Figure 4E). Thus, the larger number of recall sessions yielded by our automated task together with the animal’s persistence to recall the rewarded port over multiple trials per session provided sufficient statistical power to reveal the traces of memories generated in previous sessions up to 72 h.

After demonstrating that the animal’s behavior showed traces of previously learned memories, we tested if we could manipulate each of these memories independently. To impair the consolidation of each memory we gave the mice an intraperitoneal injection of the NMDA-receptor antagonist 3-(2-Carboxypiperazin-4-yl) propyl-1-phosphonic acid (CPP) 30 min prior to the learning session (Kentros et al., 1998). We adjusted the dosage of CPP (5.5 mg/Kg) to an amount that did not affect the performance during learning sessions (Supplementary Figure 9) in order to affect only memory consolidation but not acquisition. By alternating days of CPP injection with a control condition (e.g., saline or no injection) we were able to separately assess the impact of CPP on the strength of memories generated 2 and 24 h earlier. When CPP was injected, it decreased the 2 h memory (*P* < 0.05) of that day but did not affect the MI from the previous day (non-significant by *t*-test) (Figure 4F, compare black vs. dark-green lines). When memory was tested the day following CPP injection, the 2 h memory was not affected (*P* = 0.41) but the memory from the day of the CPP injection was decreased (*P* = 0.08) (Figure 4F, black vs. light-green lines). A significant interaction between *drug* = {CPP today, CPP day before, no CPP} and *lag* = {2 and 24 h} was shown with a two-way ANOVA (*P* < 0.022), indicating that the decrease in MI caused by the CPP affected only the memory of the day of the injection, and not memories acquired in control conditions. These results show that in our task blocking NMDA-r with CPP caused an impairment of the consolidation of a newly formed memory without affecting previously-stored memories or the consolidation of memories in upcoming days.

### Neuronal Activity Recordings While Animals Perform the 8-Port-Maze Task

**FIGURE 5:**
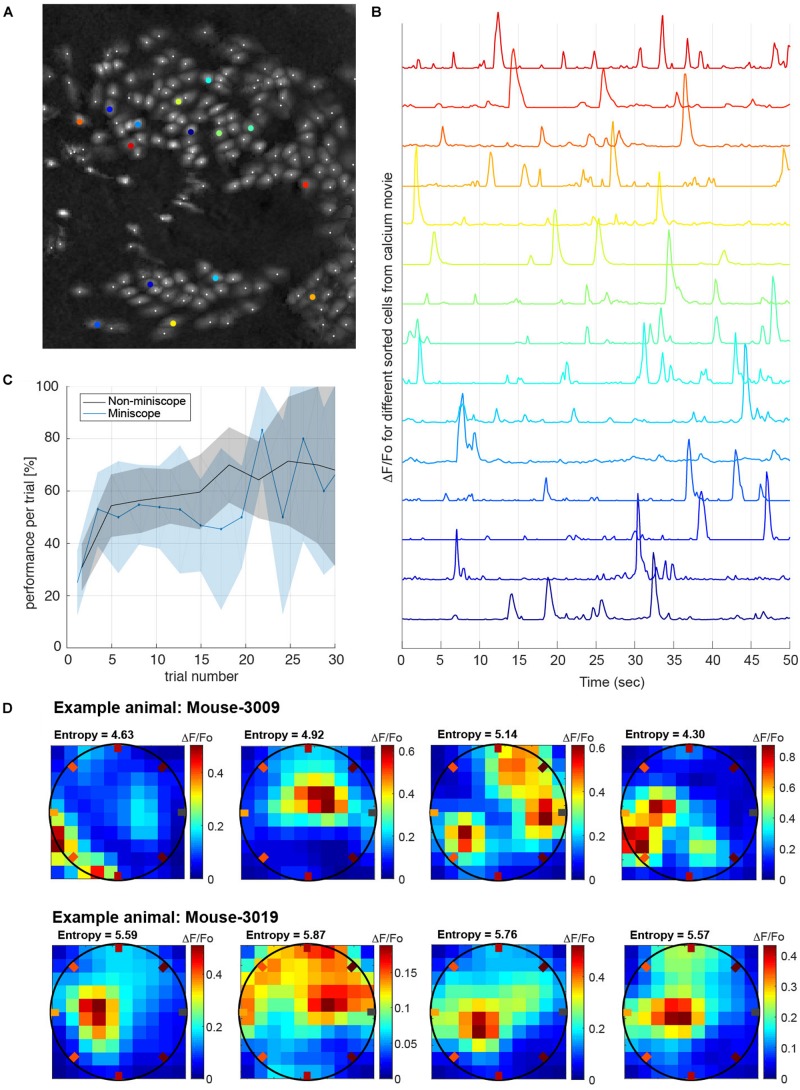
Calcium imaging recordings while animals perform the task. **(A)** Example frame calcium imaging registration of the cells recorded simultaneously while the animal is performing the task (approximately 180 neurons recorded). **(B)** Calcium activity traces of relative fluorescence (ΔF/F_o_) recorded from selected neurons (colored in **B**). Traces were processed off-line and used to decode different behavioral tasks (e.g., animal’s position). **(C)** Performance measure in interleaved sessions with and without mini-microscope. The performance versus trial number does not show a significant difference if we compare the conditions with the animal carrying the mini-microscope (*P* = 0.5, one-tailed *t*-test). Shaded areas are the mean’s 99% conf. interv. **(D)** Example place-fields obtained from two different animals while performing the task. Place-fields were chosen from the distribution of spatial information measured by the entropy of the place-fields neuronal tuning (Markus et al., 1994). All recorded place-cells have entropies higher than 2.5 bits/sec.

Having shown that our task can quantify the behavioral traces of multiple memories learned over a few days, we wanted to demonstrate, as a proof of principle, that animals were able to perform the task while their neuronal activity was recorded. We implanted miniaturized-microscopes (nVista) in two animals and recorded the calcium activity from up to 300 neurons in the hippocampal area CA1 during 15 learning and recall sessions (Figures 5A,B). Performance during sessions in which the two mice carried the mini-microscopes was not different from sessions in which they were untethered (Figure 5C). Utilizing analyses performed offline, we re-aligned frames from the mini-microscope recordings to overcome the deformation suffered by the individual images due to brain movement when the animal ran during the task (Pnevmatikakis and Giovannucci, 2017). The selection of cell body boundaries to extract calcium activity from individual neurons were obtained using an EM algorithm (CELLMAX, Kitch, 2015). An example of the spatial layout of the neurons sorted from the calcium activity movies while the animals performed the task is shown in Figure 5A. After preprocessing of the movies, we obtained traces of calcium activity from each individual neuron (Figure 5B) that were matched with the animal’s position and poke timestamps during the task, to study correlations between neuronal activity and behavior. Given the dense spatial coverage obtained in the behavioral task, we were able to characterize neuronal tuning such as place-cell response patterns (see section “Methods”). As an illustration of the quality of the neuronal data, we plotted the response from several neurons during one of the learning sessions (Figure 5D).

## Conclusion

We present a novel memory task that allows robust measurement of several memories stacked over time with the following characteristics: (1) a complete automation of the task; (2) a trial-based structure in which memory recall was repeatedly probed over the course of several months; (3) randomization of the initial position of the animals, forcing them to perform different trajectories in each trial and generate uniform spatial coverage; (4) overt recall responses in the form of precisely timed nose pokes allowing the study of multiple memories stacked over time; (5) well-defined measurement of memory accuracy (i.e., the MI) that allows comparison among different experimental conditions.

The majority of available tasks aimed at studying memory recall over days were not designed for high-throughput experimentation which is key to establish correlations between neural activity and behavior (Table 1). One of the first studies that was able to show a correlation between memory and neuronal activity using recordings from hippocampal neurons during a spatial navigation task in an open field was conducted by Kentros et al. (2004). Despite the importance of the results, a metric to relate neuronal activity with the accuracy of the animals solving the memory task was lacking. Another important contribution to the study of the neuronal representation of spatial navigation was developed by Dupret et al. (2010). In this study animals learned the location of three rewarding wells in a lattice of wells, allowing the quantification of the memory strength. Animals learned to navigate from their “home” station to the rewarded wells over 40 trials per session, and the memory accuracy of the rewarded locations were tested on a single trial memory recall session, 2 and 24 h later. The task allowed researchers to collect neural activity, but the assessment of changes in neuronal tuning related to learning was constrained due to the lack of spatial coverage and overt responses in a structure of trials. Pfeiffer and Foster (Pfeiffer and Foster, 2013) carried out a study that related the hippocampal neural representation of space with future planning. In each trial, animals learned to shuttle between a fixed location rewarding well and a randomly located rewarding well. Animals repeated the task for 30 trials on two consecutive days. Memory recall was not measured for periods longer than 200 s, and thus this paradigm did not assess long-term memories. The task was also supervised to detect when a well was emptied by the animal, at which time it was refilled by the experimenter. These behavioral paradigms have three main limitations: (1) labor-intensive human intervention, which prevents running multiple parallel experiments; (2) the relatively small spatial coverage during the task, which hampers obtaining a good characterization of the spatial selectivity of neural activity (e.g., place-fields); and (3) the lack of cue triggered overt memory test responses (for comparisons see Table 1).

**TABLE 1:** Comparison of other spatial navigation memory tasks.

	Allows neuronal activity recordings	Short or non-pre-training required	Trial structure during memory test	Stimulus triggered overt memory recall responses	Provides large numbers of memory recall trials	Suitable for a large number of sessions	Fully automatized (computer controlled)
Context memory by foot-shock conditioning	✓	✓	×	✓	×	×	✓
Morris water maze (Steele and Morris, 1999)	×	✓	✓	×	×	×	×
8-radial arm maze	✓	✓	✓	×	×	✓	×
Barnes maze	✓	✓	×	×	×	✓	×
Spatial object recognition (SOR or NOL)	✓	✓	×	×	×	×	×
Virtual water maze (Kentros et al., 2004)	✓	×	✓	✓	N/A	×	✓
Cheeseboard food wells (Dupret et al., 2010)	✓	×	×	✓	×	✓	×
Cheeseboard food wells (Pfeiffer and Foster, 2013)	✓	×	✓	×	N/A	✓	×
8-port maze task	✓	×	✓	✓	✓	✓	✓

The automation of our novel task facilitates a high-throughput behavioral approach (Aoki et al., 2017; Han et al., 2018), allowing one experimenter to run several animals in parallel in different boxes (Supplementary Figure 1). Unsupervised behavioral testing reduces the costs of human intervention, decreases the manipulation of animals, presumably minimizing stress, and has the advantage of removing the experimenter’s subjective component, both during training and when quantifying behavior. Moreover, behavioral automation requires the standardization of training protocols (e.g., setting well-defined criteria for when animals must progress in the training), ultimately increasing the overall reproducibility of the data. Our design should be viewed as an important step towards fully automated behavioral control and monitorization in a novel task. Future implementations should consider self-paced sessions, where animals could voluntarily access the arena from their home cages to perform the task when they have the necessity to drink water (Rivalan et al., 2017). Furthermore, memory recalls could then be automatically scheduled at different time delays.

Behavioral assays in mice are typically hindered by the large and unexplained variability exhibited by animals during and across repeated experiments. During recall sessions, our mice also exhibited variability in their search behavior (Figures 3A,B): they tended to poke multiple ports in seemingly random order. By structuring recall sessions in a sequence of repeated trials, we overcame this intrinsic limitation and were able to obtain adequate statistical power. Motivation across sessions as a source of variability was minimized by making the animals self-pace the trials, having to actively search for the trigger zone. During the recall session, to maintain the motivation over multiple trials required the creation of conditions in which animals were uncertain about when the reward would be available. This was done by randomly changing the length of the no reward period in recall sessions from 0 to 5 min. Had we maintained a fixed duration of the no reward period, mice would have learned that there was no gain (reward) to perform the task during that period, resulting in fewer trials. We found that this was not the case in our experiments, and animals kept attempting to harvest reward in the recalled port with maintained persistence (Figure 3J). Moreover, having the possibility of running the experiment over dozens of concatenated sessions, not only allowed us to achieve substantial statistical power in the quantification of memory accuracy (Figures 3, 4) but opened the possibility of studying a constellation of questions regarding the interactions between memories. For example, can stored memories be swapped during the recall, can the trace of a newly formed memory be affected (e.g., spatially shifted) by the existence of similar memories, is there a relationship between the acquisition of new memories and forgetting old ones? Moreover, using the task in pharmacological models (e.g., NMDA receptors blockers) or in other animal models of inducible amnesia, we could further dissect the mechanisms at play during the recall of memories stacked across time.

In addition to the development of a behavioral task that allows studying multiple memories stored on previous days we have also provided statistical and mathematical analysis tools for the unbiased measurement of such memories. Indeed, we were able to quantify the statistical strength of memories of previous days by developing the MI and the corresponding non-parametric test of significance using confidence intervals from surrogate data, on a session by session basis. We pharmacologically altered the formation, consolidation, and recall of the memories and determined the effect of these manipulations on memories with different time lags.

Finally, our task required animals to learn to poke in the different ports to obtain the reward, in contrast to other tasks that make use of natural behaviors that do not need training. Once animals learned to perform the task (which typically took 20–28 computer-controlled training sessions), the behavioral output was delimited to a discrete series of overt responses, between the initiation of the trial (sound trigger) and the end of the trial (nose poke in the correct port, or the end of the tone). This feature not only facilitates the interpretation and analysis of the data because pokes are precisely timed, but also allows for the synchronization of brief and precisely timed optogenetic manipulations, inhibiting or exciting different brain areas at precise times around these memory recall events. Moreover, obtaining a well-timed behavioral output facilitates the use of electrophysiological or calcium-imaging recordings aimed at characterizing the neural correlates underlying the generation of memory-recall processes. In sum, this novel memory task is a powerful instrument to investigate the processes of memory formation and recall and to dissect the neural circuit dynamics underlying these functions.

## Methods

### Behavioral Box

The arena was a Hexadecagon with 70 cm diameter, made of exchangeable rectangular vertical panels (Figure 1A), and with a standard water port (mouse port assembly, Sanworks, LLC) located in every other panel. The eight ports were connected to the computer via an Arduino “Mega,” using the serial port. We collected data from the ports at approximately 100 Hz (10 ms interval between samples). A GUI in python displayed all behavioral readouts in real-time for performance monitoring during learning and recall sessions. During pre-training, the GUI also gave the alarm when the criteria were reached to pass the animals to the next stage (see *pre-training* below).

### Behavioral Paradigm – Learning Session

All animal procedures were approved and carried out in accordance with institutional guidelines (Generalitat de Catalunya: Autorització de projecte d’experimentació *N*° = 9997), and the Comitè Ètic d’experimentació Animal (CEEA) at the University of Barcelona, *N*° = 121/18. We used C57BL6/J male mice (Jackson Labs; 9–10 weeks old) that were water restricted (0.5 mL twice a day) for 1 week prior to pre-training, resulting in a drop to 80% of their starting weight. During the pre-training animals recovered to ∼95% of their original body weight by only drinking during the two daily sessions. Experiments were run 6 days per week with some animals tested for up to 240 days, without signs of stress or dehydration. The starting time of the experiments for each animal was random in order to average out circadian rhythm effects on learning performance and memory.

Animals were introduced in the arena by the experimenter, who also started the program controlling the behavior. The program recorded the behavior, tracked the position of the animal in real-time, and collected the nose pokes in each of the eight ports, and recorded the timestamp of the calcium frames (or the electrophysiology TTL) when these recordings were acquired. Mice had 1.6 min (100 s) for acclimatization after they were introduced in the arena. After that, the first trigger zone (a disk of 17.5 cm diameter, 1/16 of the arena’s surface) was generated and placed randomly (with a uniform distribution in the circle) within the limits of the arena. Animals were trained to find the rewarded locations while the sound cue was active (maximum 6 s). Once the animal found the correct port, they remembered its location during the rest of the session (making few mistakes sporadically, see raster Figure 2A). Once the animals collected the reward, they started moving in random trajectories within the enclosure to trigger a new sound cue that signaled when the reward was available at the correct port. The sessions lasted for 15 min beyond the acclimatization time, and animals made between 30 and 100 trials, without influencing the willingness to obtain the reward during recall sessions (see Supplementary Figure 10).

### Behavioral Paradigm – Recall Session

Animals were introduced in the arena by the experimenter and had 1.6 min for acclimatization. After that period, the first trigger zone was randomly generated within the limits of the arena as in the learning session. Water was not available during the recall session for the first 0, 1, 3, or 5 min depending on the day, randomized and also based on the performance and motivation of previous days’ recall sessions. The sound cue was not interrupted when the animal poked the correct port, to avoid the feedback signal that could have interfered with the memory recall. The correct port was the same as in the prior learning session, performed 2 h earlier on that same day. After the period with no water, the recall sessions became identical to the learning session, reinforcing the memory of that port. The recall session lasted 10 min. beyond the acclimatization period.

### Behavioral Paradigm – Pre-training

This period took 22.3 days [range: 20–28], during which animals performed one or two sessions a day. In general, after two consecutive days with performance higher than 90%, animals were passed to the next stage. The same criteria were used for every stage of the pre-training sessions. On the first day, there was a cue light on the correct port during the tone in all trials. Only on the first day, there was a small water drop hanging from the water spout on the correct port once the sound cue started. The reward was obtained when the animal poked the correct port during the tone (Figure 1B). The sound cue lasted for up to 220 s. After approximately 2 days animals achieved above 90% performance and they were passed to a new stage. This new stage had light cueing the location of the correct port only for the first two trials and lasted between 2 and 3 days. Then, the sound cue was reduced to 20 s in each trial. At this stage, animals reached criteria after 5–10 sessions. On the next stage, there was no light indicating the correct port and in general, the criteria were reached after 5–6 sessions. In this stage, we required a minimum of 30 correct trials per session allowing us to obtain large data sets needed to correlate neural activity with behavior. The next stage was the last where the learning session started: sound cue lasted up to 6 s. Once they reached criteria and had more than 30 trials correct on learning sessions, animals started to be tested on the recall session 2 h after the learning session on the same day.

### Analysis of Behavioral Data

The output data for each session consisted of a full video of the session recorded from a camera located above the maze, the trajectory of the animal during the entire session, the locations and times of the trigger zones, and the poke times for every port. These data sets were analyzed offline to characterize behavioral performance and to correlate behavioral variables with calcium imaging activity. A poke was defined as an entry into a port recorded by the interruption of an infrared light bin. We did not consider consecutive pokes in the same port unless the animal had moved 5 cm away from that port. The poking times were also saved to represent the persistence of each poke (shown as small dots in Figures 2A, 3A), but these pokes were not included in the analyses. Four out of twenty-six animals used for the learning sessions were not included in the analyses, because their average performance across learning sessions did not reach a steady-state above 85%. These four animals did, however, learn the task with performances higher than 50%. The exclusion of lower-performing animals was to maximize the possibility of correlating neuronal activity with behavior by obtaining high numbers of correct trials. We only included recall sessions in the analyses with a minimum of three completed trials, established as a condition on task engagement. We quantified the accuracy of the memory recall using the memory index (MI) which was computed in three conditions: (1) 2 h MI for single sessions and single animals (Figures 3F,G,I); (2) 2 h MI of single animals averaged across all recall sessions (Figures 4A,E and Supplementary Figure 7); (3) MI of previous days averaged across sessions and animals (Figure 4 and Supplementary Figure 8). To compute the MI of single sessions, we first computed a poke histogram of the session representing the total number of pokes *N*_i_ in each port. The port index *i* = −3, −2, −1, 0, 1, 2, 3, and 4 labeled the different ports with respect to the correct port *i* = 0 chosen to provide reward that day. We then normalized each histogram *n_i_* = *N_i_/N*, where *N* was the total poke number in that session *N* = *N_1_* + *N*_2_ + …*N*_8_. Finally, we computed the *poke vector V* that measured the net excess of poking probability (vector length) toward one spatial direction (vector angle) (see black arrow in Figures 3F,G). The vector was defined as the vector sum of the eight subvector components of length *n*_i_ and angle θ*_i_*, each representing the tendency to poke the corresponding *i-*th port. The angles of the ports were θ*_0_* = 0, θ*_1_* = π/4, θ*_2_* = π/2, θ*_3_* = 3π/4, θ*_4_* = π, θ*_–3_* = 5π/4, θ*_–2_* = 3π/2 and θ*_–3_* = 7π/4. Thus, the x and y components of *V* were precisely defined as:

$$V_{X}=\sum_{i=1}^{8}n_{i}\,\cos\left(\theta_{i}\right),V_{Y}=\sum_{i=1}^{8}n_{i}\,\sin\left(\theta_{i}\right)$$

The memory index was equal to the x component of the pokes vector, MI = *V*_X_ (see the red segment in Figures 3F,G,I). To compute the average MI across recall sessions in a single animal, we first created a pooled poke histogram by summing the poke histogram of each session (always aligned at the correct port; Figures 3F,G), normalized the pooled histogram and then obtained a session-averaged MI (red segment in Figures 3F,G), just as we did from the single session poke histograms. To obtain the MI of the rewarded ports learned on a previous day (e.g., day lag −1, −2, …−8), we realigned each single session histogram with respect to the port that had been correct on the previous day. Sessions from the same animal were then pooled, normalized and averaged across animals (Figures 3H,I). MIs for the memory of previous days (e.g., day lag 0, −1, …−7) were then obtained from these histograms as described above (Figure 4E and Supplementary Figure 8).

To assess the statistical significance of the poke histograms and MIs we generated surrogate data sets representing poking activity compatible with the null hypothesis, and obtained 99% confidence bands. For single sessions, we generated *M* = 500 poking surrogate sets, each generated from a uniform distribution of pokes across the eight ports (e.g., there were no port reference) and having the exact same total number of pokes *N* as the original data from that session. For each surrogate set, we obtained the poke histogram and the MI as done with the original data. We then obtained a *P* < 0.01 pointwise significance bound for the poke histogram (magenta dashed line in Figures 2D, 3F,G,I, 4A and Supplementary Figure 7) and for the MI (magenta dashed line in Figure 3J, 4E and Supplementary Figure 8 magenta mark). For the session-averaged histograms, we adopted a more conservative null hypothesis: surrogate data sets were obtained by shuffling the port indices of the single session histograms, then pooled over sessions, normalized and converted into MIs. This shuffled data represents a null hypothesis in which the poking distribution deviates from a uniform distribution similar to the original data (e.g., certain ports were preferred over others in each session), but these preference deviates show no relation with whether the port was correct or not. Finally, assessing the significance of memories from previous days required the generation of surrogates from a generative model representing the null hypothesis. As the location of the correct port was not independently drawn in each day and for each animal, we minimized the probability that (1) two animals running on the same box had the same correct port on the same day, and (2) that an animal repeated the same correct port on consecutive days. The second condition introduced serial correlations in the sequence of correct ports experienced by each animal across days. In particular, the probability that today’s correct port had been the correct port on previous days was lower than chance (see Supplementary Figure 8a). This results in an increase in the probability to poke today’s correct port, which would automatically cause an artificial lack of probability to poke on the correct port from previous days. To address this problem, we modeled the poking behavior of an agent who only had a memory about the correct port of the same day (e.g., only 2 h memory) with the MI matching the MI of every single session original data. In particular, the distribution of pokes of the model was uniform in all ports except the correct port in which there was the precise excess (or lack, in case of negative MI) of probability to match the MI of that day. We used this model to generate *M* = 500 surrogate sets for each session and each animal, using the same number of pokes and following the exact same correct port sequence as in the experiment. From the M surrogates, we obtained averaged histograms for the memories of previous days. These histograms showed a trough in the center corresponding to the lack of poking probability caused by the spurious interaction of the 2 h memory across days (see red dashed lines in the lower insets of Figures 4B–D). Because of this property, the MIs for previous memories obtained from these surrogates were negative for the immediately preceding days and then tended to zero as the session lag increased (gray line in Supplementary Figure 8b shows the surrogate mean MI) due to the shape of serial correlations among correct ports across days (Supplementary Figure 8a). To simplify the display of MI versus memory-lag we discount from the MI for different lags, the corresponding value of the surrogate mean MI (Figure 4E). We finally obtained the significance bound (*P* < 0.01) from this surrogate MIs and used it to assess the significance of the original data MI (comparison between black dots and the red dashed line in Figure 4E). We checked that this significant bound did not change if the null hypothesis was modeled using a Von Mises distribution for pokes.

### Two-Way ANOVA to Test the Effect of CPP on Previous Memories Recall

Animals received an intraperitoneal (IP) injection of CPP to reduce the consolidation of a memory acquired during the learning sessions. The memories were tested by measuring the MI relative to the correct port for the given memory lags (0, 1 day). We built a two-way ANOVA to test if the presence of CPP could explain the variance of the data. The ANOVA matrix was built with the corrected MI and two classes; the type of data set {type 1 = [NO-CPP on lag 0, NO-CPP on lag 1]; type 2 = [CPP on lag 0, NO-CPP on lag 1]; type 3 = [NO-CPP on lag 0, CPP on lag 1]}, and the lag: 0 or 1. The two-way ANOVA performed on the data in Figure 4F showed a significant correlation with the memory lag *P* = 2 × 10^–10^, with the effect of CPP on the MI change *P* = 0.004, and most importantly the interaction term between CPP and lag was *P* = 0.022. We also tested individually if the effects of CPP on each memory test at 0 or at day 1 lag were significant by using a one-tail *t*-test for two different size populations, where the null hypothesis was that they belong to the same distribution.

### Pharmacology

For experiments with CPP animals received an IP injection of 0.5 mL CPP diluted in saline or saline. We initially tested multiple doses ([4.5,5,5.5,7,8.5,9,10] mg/kg) and found that 5.5 mg/kg was the optimal dose with minimal effect on behavior (no change in locomotion and performance during learning sessions and rewarded phase of recall sessions) and maximal effect in disrupting memory. For experiments with muscimol and artificial cerebrospinal fluid (ACSF), we injected a 100nL bilaterally through intracranial cannulas (Plastic One system) aiming for the dorsal hippocampus (coordinates: AP: −1.8 mm, ML: ± 1.8 mm, Depth from dura: −1.1 mm). We tested several doses ([0.3, 0.4, 0.5, 0.6, 0.66, 0.7, 1] μg) and determined that 0.66 μg was the highest dose that did not produce changes in locomotion (e.g., total distance covered and mean speed). Injections were done utilizing Hamilton syringes and Harvard apparatus pumps. The location of the cannulas was ± 1.8 mm from the middle line on each hemisphere, 1.8 mm rostrocaudal from bregma, and 0.8 mm, deep. Between experiments, to keep the brain free of infections, cannulas were filled with a dummy cannula, with a protrusion of 0.5 mm. The injection of muscimol and ACSF lasted 3 min. with a 7 min. resting period with the injection cannula inserted to avoid leakage. Animals were held by their implanted head-bars and attached to a running plate to reduce stress.

### Surgeries for Implantation of Mini-Endoscopes for Calcium Recordings

Animals received food and water *ad libitum* for 2–3 days prior to the surgery. All animal procedures were approved and executed in accordance with institutional guidelines (Generalitat de Catalunya: Autorització de projecte d’experimentació *N*° = 9997), and the Comitè Ètic d’experimentació Animal (CEEA) from University of Barcelona, *N*° = 121/18.

### Viral Injection

Surgeries were performed when mice were between 16 and 18 weeks of age, once they were trained on the task and their average performance across days reached 80%. We label excitatory neurons by injecting an adeno-associated virus (AAV, serotype 2.5) driving expression of GCaMP6m via the CaMKIIα promoter. Mice were anesthetized with isoflurane (induction, 5%; maintenance, 1–2%) in 95% O_2_, and then fixed in a stereotactic frame (Kopf Instruments). Body temperature was kept at 37°C using a temperature controller and a heating pad. AAV (600 nL) was injected via a borosilicate glass pipette with a 50 μm diameter tip using short pressure pulses applied with a picospritzer (Parker) coordinates relative to bregma in three locations: [mediolateral (ML) = 1.8., anterior–posterior (AP) = −1.5, dorsoventral (DV) = −1.6; ML = 1.4., AP = −2.2, DV = −1.55; ML = 2.1., AP = −2.9, DV = 1.8, from bregma].

### Mini-Endoscope Implantation

30 days after AAV injection a second surgery was performed to implant a mini-endoscope. This is a stainless steel guide tube (1.2 mm diameter) with a custom glass coverslip glued to one end (0.13 mm thick cover glass, Paul Marienfeld GmbH), which holds a GRIN lens which is used to focus the mini-microscope used for calcium recordings (see below). To ensure a stable attachment of the implant, once the surface of the cranium had dried six small screws (18–8 S/S, Small parts) were inserted on top of the cerebellum, olfactory bulbs and somatosensory sensory cortex to increase torque resistance of the implant. The skull was perforated with a dental milling bit of 0.7 mm diameter, and the screws inserted for approximately 0.5 mm to avoid piercing the dura. The mini-endoscope was then inserted at the position and angle that covered most of the flat area of the dorsal part of the hippocampal CA1 region [relative to bregma ML = 2.1(+ 77° on the coronal plane), AP = −2.2, DV = −1.1(from dura)]. To perform the implantation, a craniotomy centered on the injection coordinates was made using a trephine drill (1.6 mm in diameter). To prevent increased intracranial pressure due to the implant, we aspirated a cylindrical volume of brain tissue equivalent to the volume occupied by the mini-endoscope. Tissue was aspirated up to the second set of fibers crossing over the CA1 area, coming from the entorhinal cortex. Each set of fibers was recognized by identifying that their orientation was ∼60 degrees from the previous layer. Next, the mini-endoscope was lowered with the manipulator of the Kopf Table and fixed to the skull using ultraviolet-light curable glue (Loctite 4305). Metabond (Parkell) was applied around both screws, the implant, and the surrounding cranium and then acrylic dental cement (Coltene, Whaledent) was placed on top of the Metabond, for the joint purpose of attaching a metal head-bar to the cranium and to further stabilize the implant. Mice return to full behavioral activity 3 days after surgery. To allow for the resolution of neuroinflammation calcium imaging was carried out 5–7 weeks after surgery. Prior to calcium imaging recordings, the level of GCaMP6m expression was checked by locating the GRIN lens within the endoscope. If the expression was sufficiently high the miniature microscope’s base-plate was mounted (nVista HD, Inscopix, Inc.) utilizing acrylic cement and the ultraviolet-light curable glue (Loctite 4305).

### Calcium Recordings During Behavior

A second computer was used to control and store the frames captured by the nVista mini-microscope. The TTL output from the DAQ board to the Arduino, recorded the time-stamps of each frame captured by the mini-microscope. Calcium activity and animal behavior readouts were combined off-line. We downsampled the raw movies from the mini-microscope prior to the processing due to computer memory constraints. The NoRMCorre piecewise linear registration algorithm (Pnevmatikakis and Giovannucci, 2017) was used to minimize the frame-to-frame displacements caused by brain movement relative to the mini-microscope field of view. Next, we took the “ΔF/F_0_” of the movies by subtracting and dividing each pixel calcium activity change (i.e., ΔF) at a given time frame by its mean activity across the field of view (i.e., F_0_). We then applied the CELLMAX extraction algorithm (Kitch, 2015), which models the way the movies arise from the underlying calcium signals, and finds the most likely set of neurons in the movie by doing maximum likelihood on this probabilistic generative model. Applying this algorithm on a temporally downsampled version of the ΔF/F_0_ movies, we obtained 600 to 1000 mask candidates for the neurons in a given session. These candidates were inspected in a semi-automated manner for calcium-like dynamics and neuron-like shapes, resulting in 300–500 simultaneous neurons per session.

### Analysis of Place Cells

Place-fields are computed as the average over the visits to a given spatial bin of the mean calcium activity within a time window (in our case 50 ms). Each spatial bin receives different visits depending on the behavior of the animal in that given session. Heat-maps in Figure 5D represent spatial tuning of individual neurons from two animals (Mouse3009 and Mouse3019), where the redder the color is on the map, the higher the calcium activity for that specific location (on average). The entropy of the place-field is estimated by the equation: $E\,n\,t\,r\,o\,p\,y=-\sum_{i=1}^{N\,b\,i\,n\,s}f\,t_{i}$ and *ft*_*i*_ is the fraction of time spent on bin “i”. Entropy is a measurement of how spatial informative are individual neurons.

The place cells displayed are the ones with entropy significantly higher than the 95% conf. inter. of the entropies computed by shifting randomly and circularly the calcium trace relative to the animal position.

## Data Availability Statement

The datasets generated for this study are available on request to the corresponding author.

## Ethics Statement

The animal study was reviewed and approved by Generalitat de Catalunya: Autorització de projecte d’experimentació No = 9997), and the Comitè Ètic d’experimentació Animal (CEEA) at the University of Barcelona, No = 121/18.

## Author Contributions

LM, JR, JD, and PJ designed the experiment. LM and PJ built the behavioral boxes and the isolation chambers and performed behavioral experiments. PJ developed behavioral control software, performed calcium imaging experiments, and analyzed the data. LM, DT, and JR helped with the analysis of the data. PJ and JR wrote the manuscript. LM, DT, and JD reviewed and edited the manuscript. PJ and JD supervised the project.

## Conflict of Interest

The authors declare that the research was conducted in the absence of any commercial or financial relationships that could be construed as a potential conflict of interest.

## References

[B1] Aoki R. Tsubota T. Goya Y. Benucci A. (2017). An automated platform for high-throughput mouse behavior and physiology with voluntary head-fixation. *Nature communications.* 8 1196. 10.1038/s41467-017-01371-0 29084948 PMC5662625

[B2] Ballarini F. Moncada D. Martinez M. C. Alen N. Viola H. (2009). Behavioral tagging is a general mechanism of long-term memory formation. *Proceedings of the National Academy of Sciences.* 106 14599–14604. 10.1073/pnas.0907078106 19706547 PMC2732837

[B3] Barnes C. A. (1979). Memory deficits associated with senescence: a neurophysiological and behavioral study in the rat. *J Comp Physiol Psychol.* 93 74–104. 10.1037/h0077579 221551

[B4] Bimonte H. A. Hyde L. A. Hoplight B. J. Denenberg V. H. (2000). In two species, females exhibit superior working memory and inferior reference memory on the water radial-arm maze. *Physiology & behavior.* 70 311–317. 10.1016/s0031-9384(00)00259-6 11006429

[B5] Chrobak J. J. Hinman J. R. Sabolek H. R. (2008). Revealing past memories: proactive interference and ketamine-induced memory deficits. *Journal of Neuroscience.* 28 4512–4520. 10.1523/JNEUROSCI.0742-07.2008 18434529 PMC6670951

[B6] Dupret D. O’Neill J. Pleydell-Bouverie B. Csicsvari J. (2010). The reorganization and reactivation of hippocampal maps predict spatial memory performance. *Nature neuroscience.* 13 995. 10.1038/nn.2599 20639874 PMC2923061

[B7] Eichenbaum H. Dudchenko P. Wood E. Shapiro M. Tanila H. (1999). The hippocampus, memory, and place cells: is it spatial memory or a memory space. *Neuron* 23 209–226. 10.1016/s0896-6273(00)80773-4 10399928

[B8] Fouquet C. Babayan B. M. Watilliaux A. Bontempi B. Tobin C. Rondi-Reig L. (2013). Complementary Roles of the Hippocampus and the Dorsomedial Striatum during Spatial and Sequence-Based Navigation Behavior. *PLoS One.* 8:e67232. 10.1371/journal.pone.0067232 23826243 PMC3695082

[B9] Han Z. Zhang X. Zhu J. Chen Y. Li C. T. (2018). High-throughput automatic training system for odor-based learned behaviors in head-fixed mice. *Frontiers in neural circuits.* 12:15. 10.3389/fncir.2018.00015 29487506 PMC5816819

[B10] Kentros C. Hargreaves E. Hawkins R. D. Kandel E. R. Shapiro M. Muller R. V. (1998). Abolition of long-term stability of new hippocampal place cell maps by NMDA receptor blockade. *Science.* 280 2121–2126. 10.1126/science.280.5372.2121 9641919

[B11] Kentros C. G. Agnihotri N. T. Streater S. Hawkins R. D. Kandel E. R. (2004). Increased attention to spatial context increases both place field stability and spatial memory. *Neuron.* 42 283–295. 10.1016/s0896-6273(04)00192-8 15091343

[B12] Kitch L. J. (2015). *Machine learning meets mammalian learning: Statistical tools for large-scale calcium imaging and the study of changing neural codes.* Stanford, CA: Stanford University. doctoral Dissertation.

[B13] Lisman J. Buzsáki G. Eichenbaum H. Nadel L. Ranganath C. Redish A. D. (2017). Viewpoints: how the hippocampus contributes to memory, navigation, and cognition. *Nat Neurosci.* 20 1434–1447. 10.1038/s41593-017-0034-8 29073641 PMC5943637

[B14] Markus E. J. Barnes C. A. McNaughton B. L. Gladden V. L. Skaggs W. E. (1994). Spatial information content and reliability of hippocampal CA1 neurons: effects of visual input. *Hippocampus.* 4 410–421. 10.1002/hipo.450040404 7874233

[B15] McKenzie S. Frank A. J. Kinsky N. R. Porter B. Rivière P. D. Eichenbaum H. (2014). Hippocampal representation of related and opposing memories develop within distinct, hierarchically organized neural schemas. *Neuron.* 83 202–215. 10.1016/j.neuron.2014.05.019 24910078 PMC4082468

[B16] Miller K. J. Botvinick M. M. Brody C. D. (2017). Dorsal hippocampus contributes to model-based planning. *Nature neuroscience.* 20 1269. 10.1038/nn.4613 28758995 PMC5575950

[B17] Morales L. Tomas D. P. Dalmau J. de la Rocha J. Jercog P. E. (2019). High-throughput task to study memory recall during spatial navigation in rodents. *bioRxiv*. 10.1101/534313 [Preprint]. PMC7243682 32499683

[B18] Morris R. G. (1981). Spatial localization does not require the presence of local cues. *Learning and motivation* 12 239–260.

[B19] O’Keefe J. Dostrovsky J. (1971). The hippocampus as a spatial map: Preliminary evidence from unit activity in the freely-moving rat. *Brain Research* 34 171–175. 10.1016/0006-8993(71)90358-1 5124915

[B20] O’Keefe J. Nadel L. (1978). *The hippocampus as a cognitive map.* Oxford: Clarendon Press.

[B21] Olton D. S. Samuelson R. J. (1976). Remembrance of places passed: spatial memory in rats. *Journal of Experimental Psychology: Animal Behavior Processes* 2 97. 10.1037/0097-7403.33.3.213 17620022

[B22] Pfeiffer B. E. Foster D. J. (2013). Hippocampal place-cell sequences depict future paths to remembered goals. *Nature.* 497 74. 10.1038/nature12112 23594744 PMC3990408

[B23] Pnevmatikakis E. A. Giovannucci A. (2017). NoRMCorre: An online algorithm for piecewise rigid motion correction of calcium imaging data. *Journal of neuroscience methods.* 291 83–94. 10.1016/j.jneumeth.2017.07.031 28782629

[B24] Post AM Wultsch T Popp S Painsipp E Wetzstein H Kittel-Schneider S Sontag TA Lesch KP Reif A. (2011). The COGITAT holeboard system as a valuable tool to assess learning, memory and activity in mice. *Behavioral brain research.* 220 152–158. 10.1016/j.bbr.2011.01.054 21310188

[B25] Rivalan M. Munawar H. Fuchs A. Winter Y. (2017). An automated, experimenter-free method for the standardized, operant cognitive testing of rats. *PloS one.* 12:e0169476. 10.1371/journal.pone.0169476 28060883 PMC5218494

[B26] Rondi-Reig L. Petit G. H. Tobin C. Tonegawa S. Mariani J. Berthoz A. (2006). Impaired sequential egocentric and allocentric memories in forebrain-specific-NMDA receptor knock-out mice during a new task dissociating strategies of navigation. *J Neurosci.* 26 4071–4081. 10.1523/JNEUROSCI.3408-05.2006 16611824 PMC6673881

[B27] Rossato J. I. Moreno A. Genzel L. Yamasaki M. Takeuchi T. Canals S. (2018). Silent learning. *Curr. biol.* 28 3508–3515 10.1016/j.cub.2018.09.012 30415706

[B28] Sharma S. Rakoczy S. Brown-Borg H. (2010). Assessment of spatial memory in mice. *Life Sci.* 87 521–536. 10.1016/j.lfs.2010.09.004 20837032 PMC6457258

[B29] Steele R. J. Morris R. G. (1999). Delay−dependent impairment of a matching−to−place task with chronic and intrahippocampal infusion of the NMDA−antagonist D−AP5. *Hippocampus.* 9 118–136. 10.1002/(SICI)1098-1063(1999)9:2&lt;118::AID-HIPO4&gt;3.0.CO;2-8 10226773

[B30] Tolman E. C. (1948). Cognitive maps in rats and men. *Psychol. Rev.* 55, 189–208. 10.1037/h0061626 18870876

[B31] Vorhees C. V. Williams M. T. (2014). Assessing Spatial Learning and Memory in Rodents. *ILAR J* 55 310–332. 10.1093/ilar/ilu013 25225309 PMC4240437

