# Supplementary material for: High-Throughput Task to Study Memory Recall During Spatial Navigation in Rodents
Source: Front Behav Neurosci. 2020 May 15;14:64. doi: 10.3389/fnbeh.2020.00064 (PMC7243682; doi:10.3389/fnbeh.2020.00064)
Supplement: Supplementary file 1 [file Data_Sheet_1.pdf]

Supplementary Figures

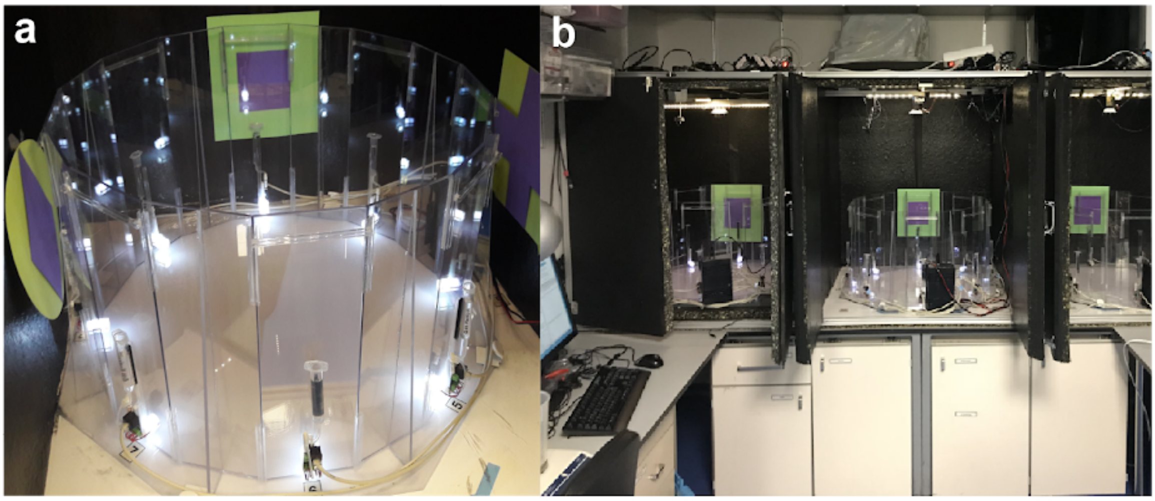

Supplementary Figure 1: Behavioral boxes for freely moving navigation tasks. a) Picture of the task maze showing the transparent acrylic walls, the water ports, and the visual cards located on the inner walls of the isolation box. **b)** picture of three sound-isolating boxes placed next to each other and controlled by the computer to minimize human intervention. With these three boxes, we were able to run 18 animals per day, yielding a total of around 1400 trials per day (including learning and recall sessions).

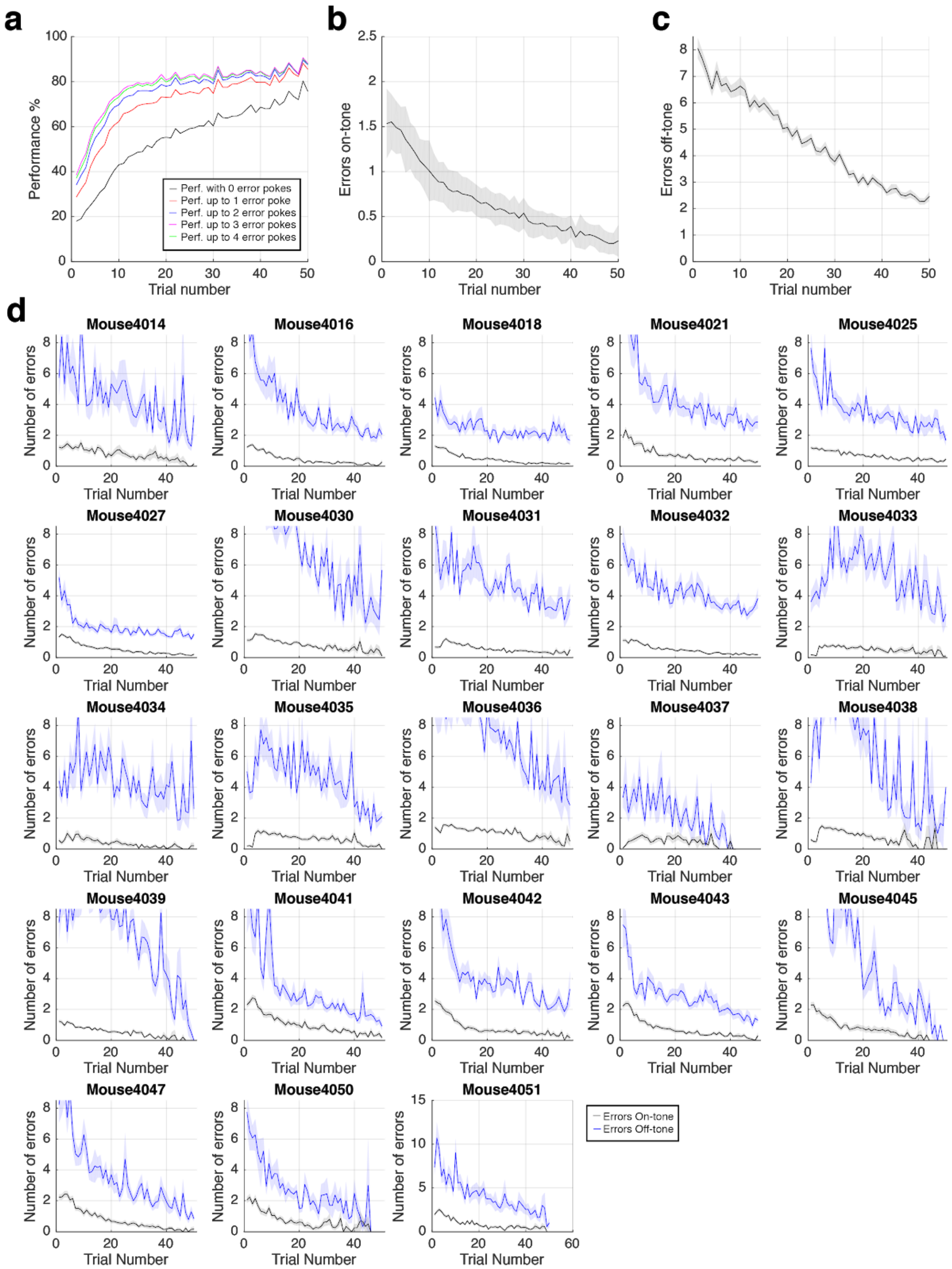

Supplementary Figure 2: Evolution of the number of errors on-tone and off-tone during the learning session. a) Performance including only trials with a different number of errors during tone, from no error to 4 errors before the poke on the correct port. No significant change in performance after including 3 errors before the poke on the correct port. The majority of correct trials are done with no or one error poke. Animals' strategy is not to poke at as many ports as possible before getting the reward. b) The number of errors during tone. On average the number of errors during tone is less than 2. **c)** Number of errors off-tone. On average the number of errors during tone starts around 5 and then decreases with the trial number. Errors off tone are due to the persistence of the animal to return to the correct port, to finish collecting all the rewards, and with trials where the animal arrived after the 6secs time window (Fig. 1b 3rd example). **d)** The number of errors on- and off-tone for each individual animal. All animals improve their performance by making fewer pokes on incorrect ports during tone, and by poking fewer ports off-tone (e.g., inter-trial interval).

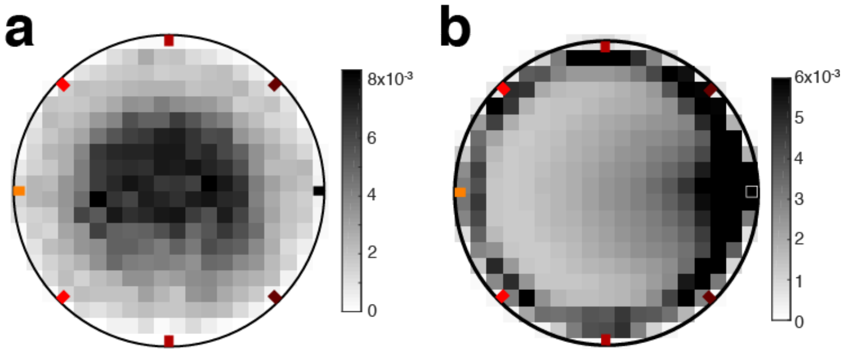

Supplementary Figure 3: Mice uniformly explore the arena to solve the task. a) Density plot of the distribution of positions where the trigger zone was intersected by the animal trajectory, for all sessions and all animals. **b)** Density map of the time spent on each spatial bin, for all animals and all sessions oriented with the correct port in the east direction (black marked port). Density is higher around the correct port, as well as on the other ports, but the coverage inside the arena is quite uniform.

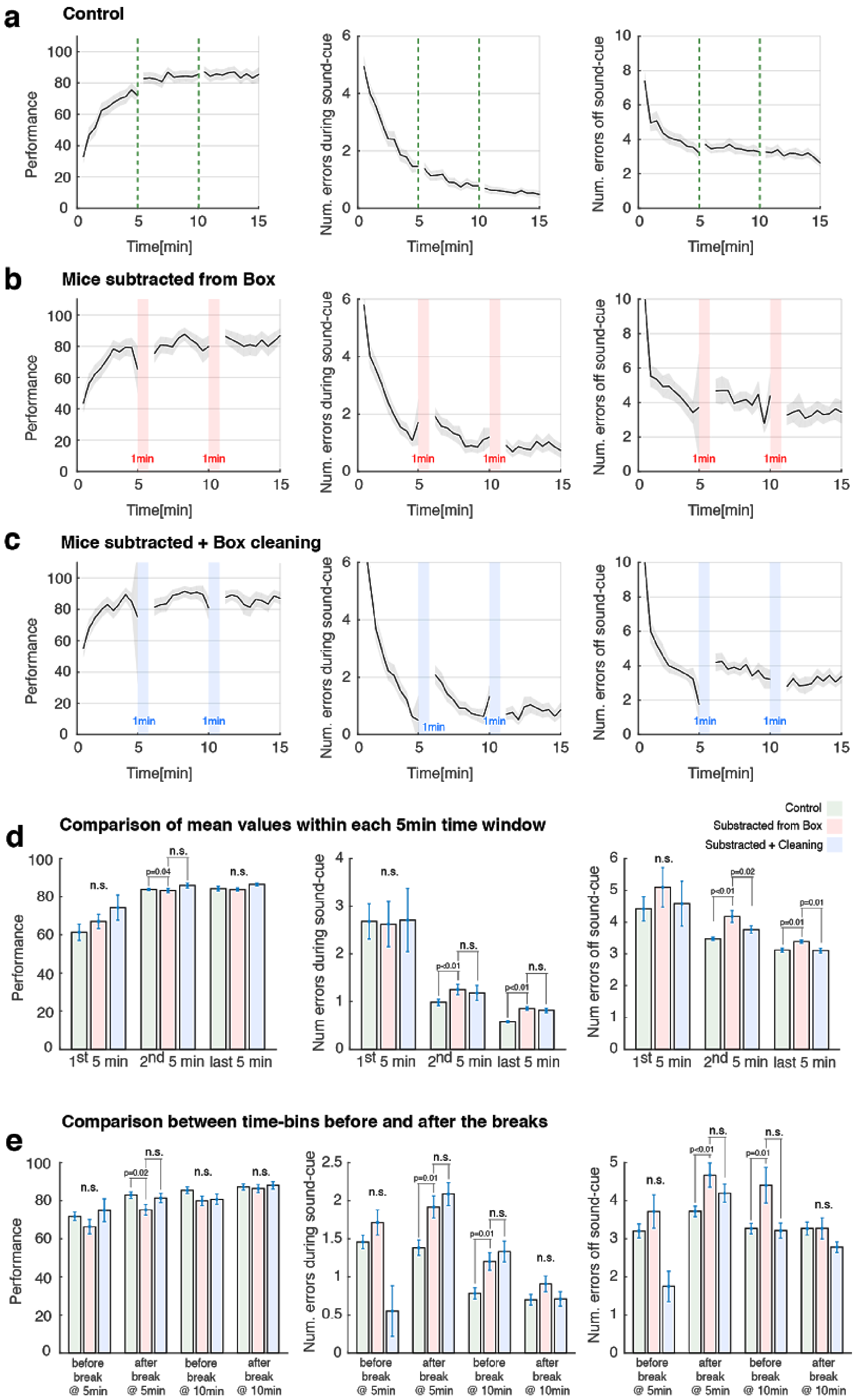

Supplementary Figure 4: No significant effect of local odors in the 8-port-maze spatial navigation task. a) Performance, number of error pokes on-tone and off-tone versus time

within-session in minutes (Control sessions). Each curve is an average of over 18 animals and approximately 20 sessions. The shaded area is the standard deviation of the grouped data on each trial number. **b)** Performance, number of error pokes on-tone and off-tone versus time within-session in minutes (subtraction-control sessions). During the 15min session, there are 2 breaks of 1 minute to remove the animal from the behavioral box, to be compared with the next set of experiments where cleaning is performed during the 1min break on all ports and the arena's surface. **c)** Performance, number of error pokes on-tone and off-tone versus time within-session in minutes (sessions with the removal of the animal and cleaning of the port). **d)** Comparing performance and number of errors between three conditions: there is no difference between data sets from subtraction and subtraction with cleaning groups in all three segments before and after 1min break, except in the number of errors off-tone. A significant difference between data sets in a) and b) are due to alteration of the task by breaks due to anticipation of the animals to their subtraction from the box by the experimenter. **e)** Similar results are observed if we only look at the 30 seconds time bins before and after the breaks.

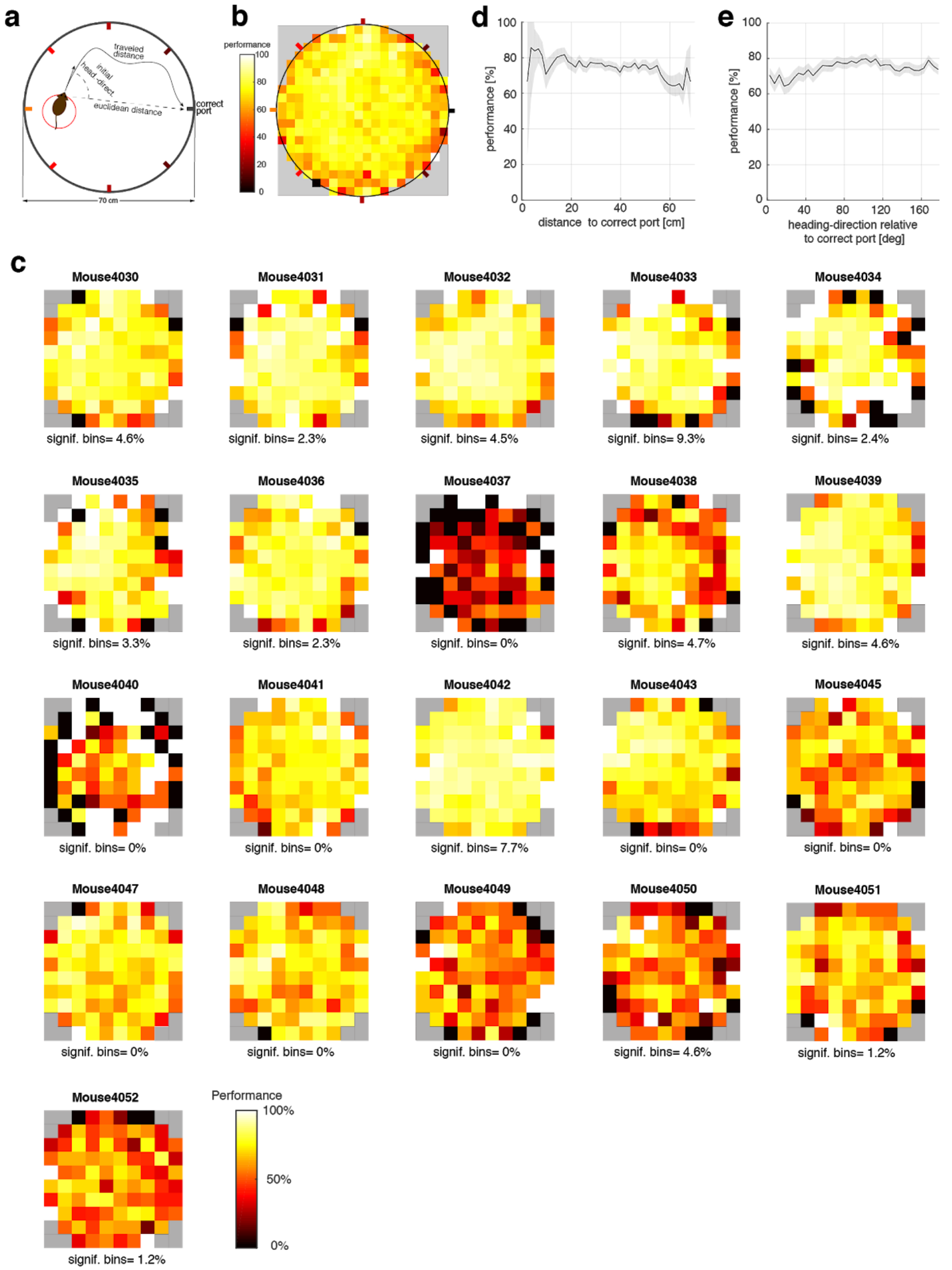

Supplementary Figure 5: Performance relative to the initial condition of the trajectory previous to the poke on the correct port. a) Schematic of the navigation to describe the initial heading direction angle and initial euclidean distance to the correct port when the animal hits the trigger zone. **b)** Map of performance outcome for a given trial, depending on the initial position on the arena. Performance does not change depending on the initial position in the arena. **c)** Each heat map represents the performance outcome for the trajectories towards the correct port started at that particular spatial bin of the map. Each map shows the percentage of bins with performance significantly higher than the shuffle surrogate data (numbers below each box). The performance is significantly different than the 95% confidence interval only on a small proportion of bins (5% is expected by chance) which we considered to not have an effect on the analysis. **d)** Performance is

independent of the initial euclidean distance from the animal to the correct port. **e)** Performance is independent of the initial heading-direction angle relative to the correct port. Error bands in all panels indicate s.e.m.

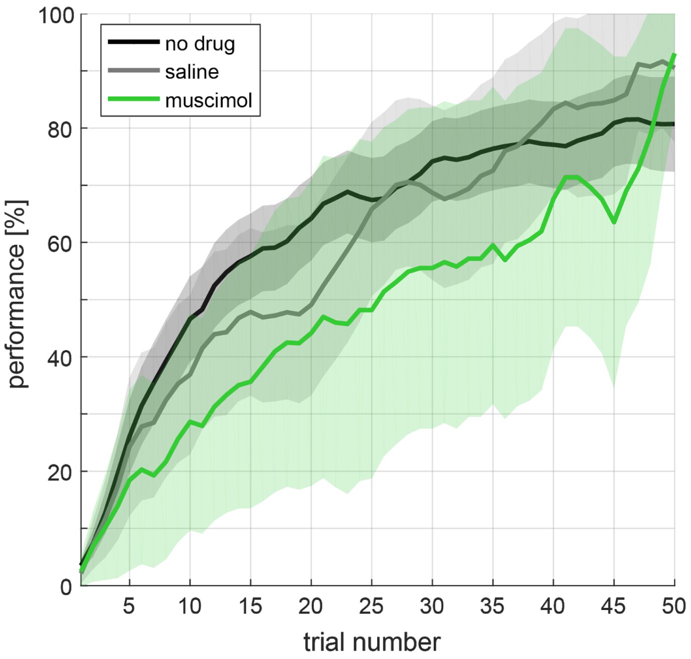

Supplementary Figure 6: Hippocampal CA1 local injection of muscimol decreased performance. Experiments were performed by bilateral intracranial injections utilizing the Harvard-pumps system of saline or muscimol (0.66ug per animal) aiming to the dorsal hippocampal area, 30 minutes prior to the learning session. The dosage selection was based on a detailed study of the dosage versus locomotion effect by measuring the distance covered and the mean speed of the animals for different concentrations of muscimol (Note: the most common dosage of muscimol for spatial navigation disruption in the literature is 1ug per animal). This protocol was repeated for a maximum of 2 weeks, alternating a day with muscimol with a day with saline. No-drug days were obtained from sessions (2-weeks) previous to the injection of muscimol in order to build a baseline of performance corresponding to each individual animal. The paired t-test between the three different conditions, taking each trial number as an individual sample was performed, and we were able to reject that muscimol was coming from a different distribution than saline ($P<0.01$) and from a different distribution than no-drug ($P<0.001$).

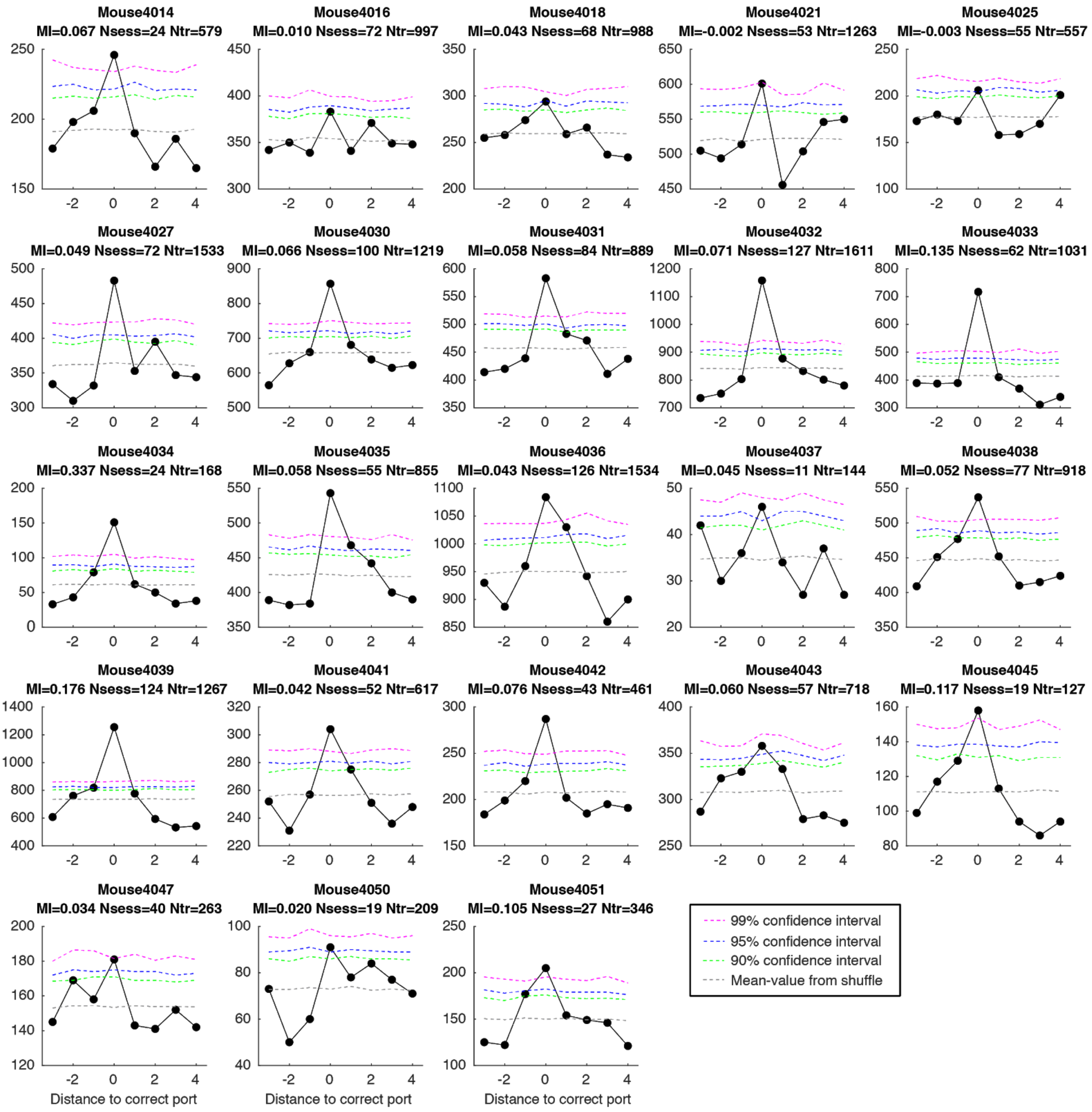

Supplementary Figure 7. Poke histograms during recall sessions for individual animals. Poke histograms of each animal (different panels) averaged across all recall sessions (see *n* above each panel; pokes were taken from both on and off sound cue periods). The correct port index was zero. Magenta dashed lines represent a 99% confidence interval (one tail) obtained from shuffled surrogate data separately generated for each animal. Blue and green correspond to the 95% and 90% confidence interval. Counts above this band are significantly different from the confidence interval. Gray dashed line is the mean value of the shuffled data. Memory indices (MI) obtained from each of these histograms are indicated above each panel, together with their degree of significance. All animals (except Mouse 4016, equivalent to 96%) express memory recall for 2 hr delay with 95% confidence. Only 65% of animals on this batch express 2 hrs memory recall with 99% confidence.

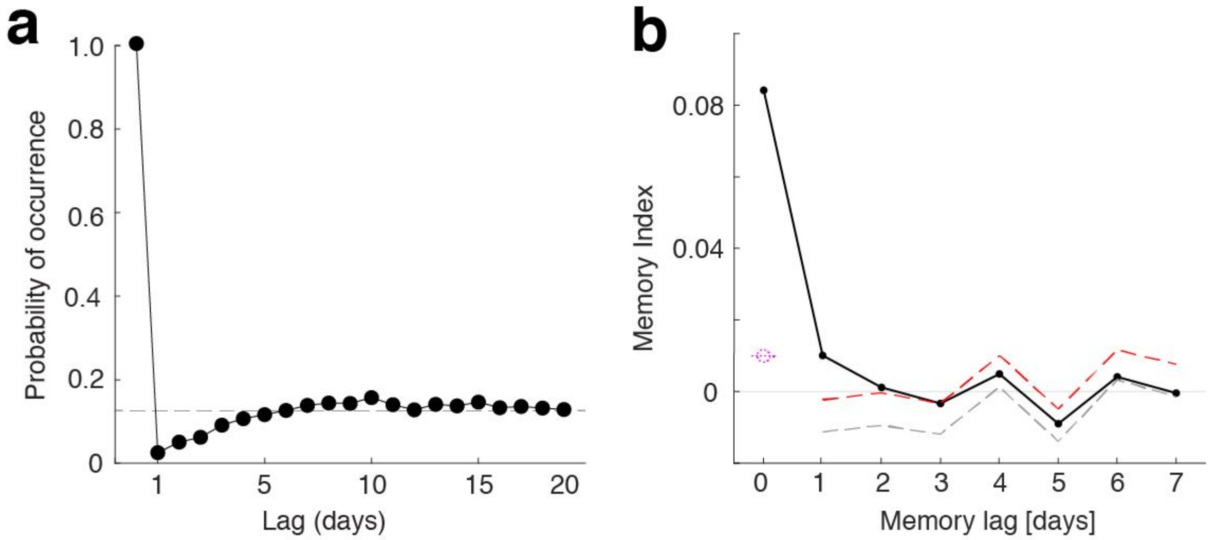

Supplementary Figure 8. Memory index for different lags. a) The probability that the correct port from a given day was also selected as the correct port in previous days. Chance level marked by the dashed line. **b)** Averaged memory index MI versus the session lag (black dots, n= 22 mice) shows significant memory recall of the correct ports from the four previous sessions (i.e. up to 72 hr, see also Fig.4a-d). The significance level for the MI at lag = 0 days (magenta symbol) was obtained from 500 surrogate data sets with uniformly sampled pokes. The significance for lags > 0 was obtained from 1000 surrogate data sets synthetically created to yield *only* the 2 hr MI found the original data (i.e. no trace of previous memories) following the same sequence of rewarded ports across days. The mean MI across surrogates (gray dashed line) was negative for the previous 3 days reflecting the lack of probability to observe the same port on consecutive days (a, see Methods). Confidence level for lags > 0 days is shown in red ($P < 0.01$).

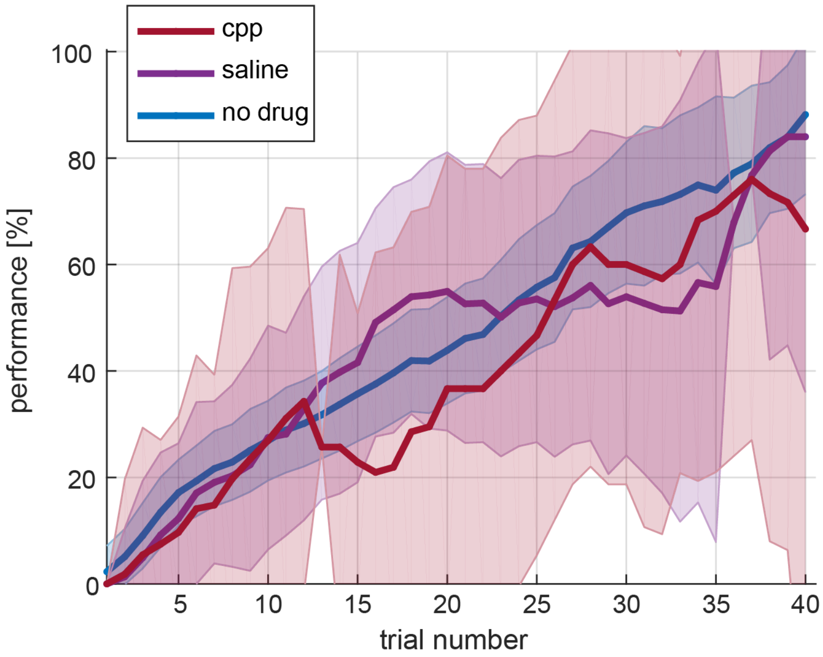

Supplementary Figure 9. CPP dosage selected for the experiment does not show a significant effect on the performance during the training session. Experiments were performed by injecting intra-peritoneal CPP or saline 30 minutes prior to the learning session. The dosage of 5.5 mg/Kg was used after studying the effect with 6 different concentrations until we found no significant effect on the behavior during learning sessions, which could affect the acquisition of memories. This protocol was repeated for a maximum of 2 weeks, alternating a day with CPP with a day with saline (in some animals alternating the sessions between CPP and saline with a no-drug day). In addition, no-drug days were obtained from sessions previous to the injection of CPP in order to build a baseline of performance for each individual animal. Based on the paired t-test between the three different conditions, taking the values of performance for each condition at each trial as an individual sample, we could not reject the hypothesis that the samples come from the same distribution (utilizing a χ^2 test).

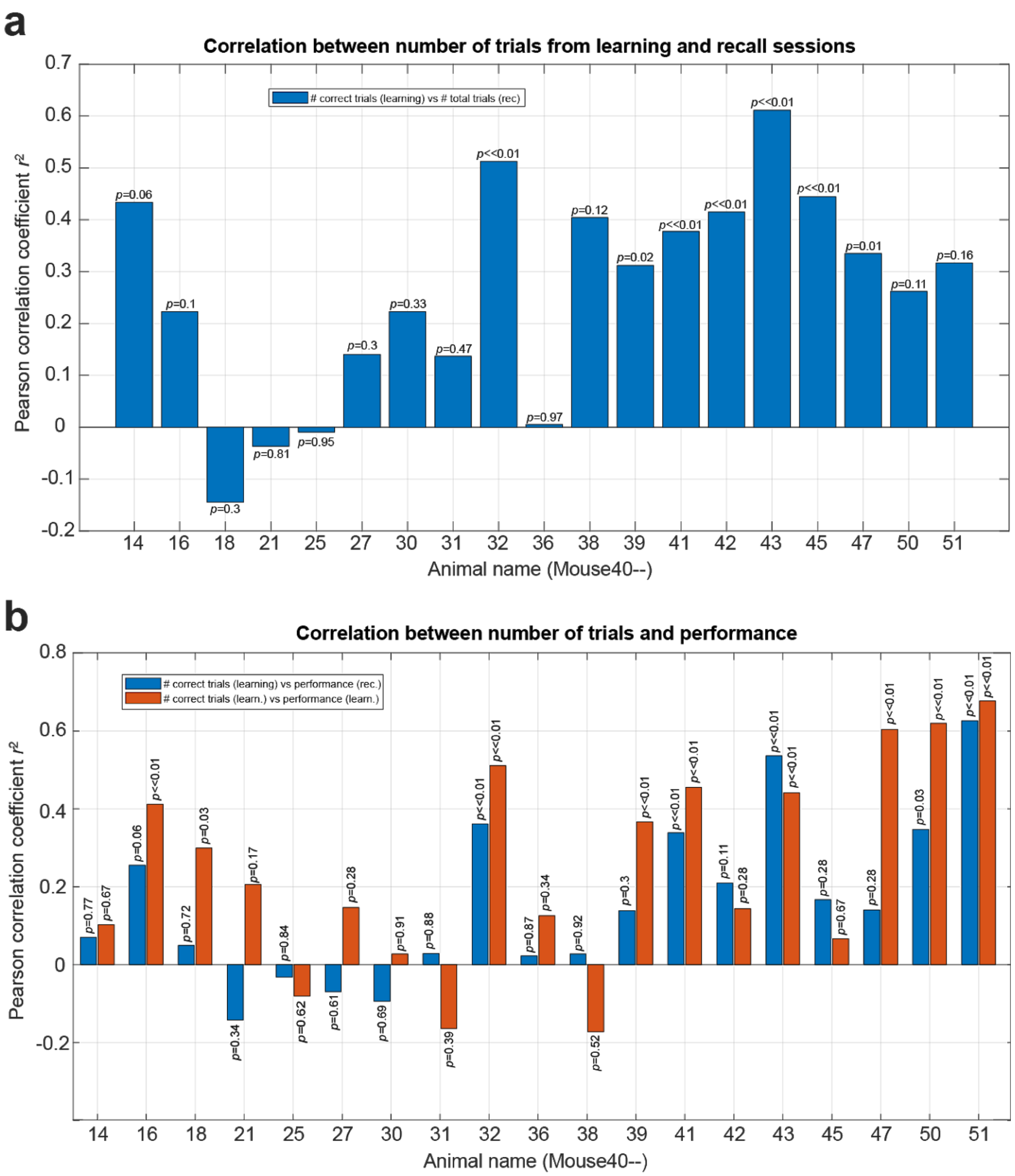

Supplementary Figure 10. Reward amount during training does not impair reward-seeking during recall. a) Correlation between the number of rewarded trials during training and total trials during recall is independent (animals with low r^2 and low p_{value}) or positively correlated. The amount of water delivered during training does not limit the reward-seeking during the recall. **b)** Correlation between the number of rewarded trials during training sessions versus the performance during recall (blue rectangles) and during training (red rectangles). We used here performance as an indirect measurement of motivation expressed by the accuracy in solving the task. As shown in this figure in the majority of animals, the larger the number of correct trials during learning sessions, the higher the performance during the recall, showing no detrimental effect of the reward obtained during the training sessions. Although counterintuitive, the correlations support the idea that the more water collected during the learning sessions, the higher the number of attempts to solve the task during the recall sessions.

Supplementary Video. Video displays the first half of a single learning session from animal Mouse4032. The video shows a circle representing the perimeter of the arena. Indicated with a small gray circle the position of the rewarding port within the arena (labeled as Correct Port). The video also displays the accumulated trajectory of the animal over the session (light gray) and a short trajectory trace of the last few seconds previous to the current animal position. The trajectory is the tracked position of the animal by our computer software. We utilized the MOG2 algorithm (e.g., Foreground Object Detecting Algorithm based on Mixture of Gaussian and Kalman Filter) to subtract the location of the center of mass of the mice from the background in real-time during the experiment. Trigger zone for each trial is represented by a red circle of 1/16 of the arena's area. Once the animal steps on a trigger zone, the sound cue starts (indicated by the text "Sound Cue On") and the animals search for the rewarding port. The sound will stop at 6 seconds if the animal does not find the trigger zone, or when the correct port is poked (black dot appears) and water is delivered. The next trial is initiated when the animal steps on the trigger zone, which could happen only after 6 seconds from the moment the previous trial sound-cue started. Soon after harvesting the reward from the correct port, the animal starts to search for the trigger zone. Pokes in incorrect ports are marked with small circles, color-coded, so the more orange the farther away is the poke from the correct port (as it is represented in all figures of the manuscript). Notice: the later in the session is the trial, the more ballistic are the trajectories towards the correct port, and the fewer the pokes in incorrect ports and outside the sound cue, as described also in the example on Fig.3a. All visual elements in this movie are stored by the software for each session.
